# A General Solution to Determine Strain Profile in the Core of Distributed Fiber Optic Sensors under Any Arbitrary Strain Fields

**DOI:** 10.3390/s21165423

**Published:** 2021-08-11

**Authors:** Xavier Chapeleau, Antoine Bassil

**Affiliations:** 1 COSYS-SII, I4S Team (Inria), Université Gustave Eiffel, Allée des Ponts et Chaussées, 44344 Bouguenais, France; 2 Quadric, 14 Porte du Grand Lyon, 01700 Neyron, France; antoine.bassil@quadric.arteliagroup.com

**Keywords:** fiber optics sensors, strain transfer, distributed strain measurements

## Abstract

Despite recent publications, the strain transfer in distributed optical fiber sensors is still often overlooked and poorly understood. In the first part of this paper, strain transfer is shown to be driven by a second-order differential equation, whether the optical fiber is embedded into the host material or surface-mounted. In this governing equation, only the value of a key parameter, called strain lag parameter, varies according to the attachment configuration and the type of optical fiber used as a sensor. Then, a general solution of the governing equation is proposed. It is an analytical expression established from new boundary conditions that are more adequate than those used previously in the literature and allows the determination of the strain profile in the core of a distributed optical fiber sensor under any arbitrary strain fields. This general solution has been validated by two experiments presented in the third part of the paper. A very good agreement between the analytical solutions and measured strain profiles using a high spatial resolution optical interrogator for both uniform and non-uniform strain fields has been obtained. These results highlight the importance of the strain lag parameter which must be taken into account for a correct interpretation of measurements, especially in the case of important strain gradients.

## 1. Introduction

For several years, there has been growing interest in new distributed fiber optic sensor technologies for both industrial and research applications [1,2,3,4]. In addition to the intrinsic characteristics of fiber optic sensors, such as lightweight, small size, immunity to electromagnetic interference and corrosion resistance, it is their capacity to provide distributed measurements of strain or temperature (in others words a multitude of measurement points along a long length of an optical fiber or optical cable) that is the most promising area for developing new innovative monitoring techniques [5,6,7]. Three main distributed fiber optic technologies, named after the backscattering of light phenomena on which they are based (i.e., Brillouin, Raman and Rayleigh that can occur in the core of the optical fiber), are currently used. Historically, distributed fiber optic systems based on Raman backscattering were first developed to measure temperature only. In the early 2000s, optical interrogators based on Brillouin and Rayleigh backscattering were then proposed to measure strain or temperature. Even today, Brillouin and Rayleigh based technologies are constantly evolving with ever more efficient measurement systems, particularly in terms of spatial resolution, maximum sensing length and measurement frequency. Brillouin systems such as BOTDR (Brillouin Optical Time Domain Reflectometry) and BOTDA (Brillouin Optical Time Domain Analysis) can reach higher sensing length than Rayleigh systems such as TW-COTDR (Tunable Wavelength Coherent Optical Time Domain Reflectometry) or OFDR (Optical Frequency Domain Reflectometry) technologies. On the other hand, Rayleigh systems are known to have the best spatial resolution, typically of few millimeters. For Brillouin systems, the spatial resolution is generally about 1 m (10 to 25 cm for the most efficient optical interrogators). Their characteristics being quite different, Brillouin and Rayleigh technologies are complementary in the sense that they cover a wide range of applications. Consequently, Brillouin systems are preferentially used to monitor large structures in the field (such as pipelines, dams, bridges, roads, railways, nuclear plants and buildings) in order to detect leakage, concrete crack, fire, intrusion, etc. Rayleigh systems are well adapted to monitor medium to small structures or specimens tested in laboratories. Contrary to Brillouin systems, it is indeed expected that the high spatial resolution of Rayleigh systems allows the measurement of local strain gradients such as those induced by the presence of a singularity (crack) or by a particular geometry or heterogeneous assembly of different materials. However, particular attention should be paid to the measured distributed strain profiles. Strain transfer is often overlooked, which can result in a misinterpretation of strain profiles measured by Rayleigh interrogators. In fact, this effect depends on the optical fiber (or cable) used as a sensing element. Given that optical fibers or optical cables used for sensing are made of several stacks of coatings with different materials surrounding the core of the optical fiber (in which light propagates), a mechanical strain transfer exists. Consequently, the strain profile in the core of the optical fiber sensor measured by a high spatial resolution optical interrogator (as a Rayleigh system without taking into account possible artifacts produced by the interrogator itself during optical signal processing) may be different to the one existing around the optical fiber sensor [8]. In order to correctly interpret strain profiles measured by Rayleigh interrogators, it may therefore be necessary to take this strain transfer effect into account.

The model of the mechanical strain transfer in a fiber optic sensor based on mathematical expressions was first developed by Ansari [9]. The fiber optic sensor was assumed to be embedded in a host material, and the geometrical model was reduced to three layers: fiber core, coating and host structure. In this model, all the components are supposed to have a mechanical linear elastic behavior. An analytical expression for the shear stress distribution was derived by Ansari based on the so-called “strain-lag” approach initially proposed in 1938 by Völkersen for the study of bonded assemblies [10] and in 1952 by Cox for the study of the behavior of a discontinuous fiber in a matrix [11]. This analytical expression is a homogeneous second-order differential equation from which a strain distribution is obtained by considering a complete strain transfer at the fiber midpoint. Although Ansari’s model is a reference in the field of fiber optic sensors, the latter assumption is not valid for short-length sensors as shown by D. Li [12]. In 2006, this author proposed an improvement of Ansari’s model by assuming that the strain gradients in the fiber core and coating are of the same order. The governing equation obtained by D. Li for the strain distribution is a non-homogeneous second-order differential equation in which a constant parameter called strain-lag parameter appears. The latter contains both the geometrical parameters and mechanical properties of the components of the three layers model. D. Li’s model was useful for evaluating the strain profile along Fiber Bragg grating sensors. In order to make an accurate measurement with this type of sensor (or at least to avoid misinterpretation of results), it is indeed important to determine the strain profile along the Fiber Bragg grating to ensure that it is almost constant in order to avoid distortion of the peak of the Bragg wavelength. By imposing a uniform strain field along the sensor by considering a symmetry with respect to the center of the grating and by assuming that the strains at the both ends of the optical fiber are null as boundary conditions, D. Li’s model permits the calculation of the strain profile in the core of the sensor and, thus, the verification that the strain transfer was quasi completed along the fiber Bragg grating. Although D. Li’s model was first developed for Bragg grating sensors, it is worth noting that nothing prevents it from being used for distributed fiber optic sensors. Later on, similar models were proposed for describing the strain transfer. For instance, Feng [13] proposed a model for distributed fiber sensors in the presence of a discontinuity in the host material. In the governing equation of D. Li’s model, he introduced the Crack Opening Displacement (COD) and obtained a mathematical expression of the crack-induced strain profile in the core of the optical fiber. He also extended the strain transfer model to include the elasto-plastic and plastic phases of the coating, as previously performed by Q. Li [14] with the Ansari model. However, in practice, the non-linear behavior of fiber optic sensors was avoided, especially for on-site monitoring, because it corresponds produces irreversible damage in the sensor. In 2016, a new improvement of D. Li’s model was proposed by Wang [15] in a study of the behavior of optical fibers embedded in asphalt pavements. Contrary to the models of Ansari and D. Li that considered perfect bonding between all the components of the three-layers system, Wang introduced an imperfect interface bonding in the strain transfer model by using an interfacial law for the interface between the optical fiber sensor and the host material. This law states that the interfacial shear stress is proportional to the relative displacement between the two layers surrounding the interface. The proportionality coefficient corresponds to the interface stiffness parameter, which tends towards infinity for a perfectly bonded interface and to zero if there is no mechanical interaction between the two layers surrounding the interface. In a study of the bonding of an embedded optical cable in concrete, Henault [16] demonstrated a from finite element analysis confirmed by experimental results of pull-out tests that the interface stiffness parameter for the interface between the embedded optical cable and the concrete substrate is an important factor to consider for proper stress transfer modeling of fiber optic sensors. It is worth noting that the governing equation of the strain transfer derived by Wang is similar to one obtained by D. Li (i.e., a non-homogeneous second-order differential equation) but with different strain lag parameter equations. Indeed, the strain lag parameter deduced by Wang additionally includes the interface stiffness parameter for the interface between the embedded optical cable and the host material. Recently, Bassil [17] generalized the imperfect bonding condition for all the interfaces of a multi-layer system, obtaining a new expression for the strain lag parameter. Then, he used this new strain transfer model for measuring crack openings in concrete [18]. A general expression of the crack-induced strain transfer from a fractured concrete material to an optical fiber was established and validated experimentally for different geometries of optical cables.

Despite these recent publications, strain transfer in distributed optical fiber sensors is still often overlooked and poorly understood. However, with the constant improvement of the spatial resolution of the optical interrogators, this effect can no longer be ignored. The understanding of the strain transfer becomes more and more necessary in order to avoid misinterpretation of strain profiles measurements. Moreover, a general method allowing the determination of the strain profile experienced by the optical fiber for any arbitrary strain distribution in the host material is still missing. Almost all publications on strain transfers are restricted to the uniform strain field in the host material. Nevertheless, it is important to underline that a recent publication [19] tried to tackle this issue by proposing, based on D. Li’s model, several formulas corresponding to various representative strain fields in the host material. However, the authors have used boundary conditions that are questionable. Indeed, they have assumed that the strain in the core of the optical fiber is equal to zero at x=0 and x=L, while they imposed a constant strain in the host material at the same abscissa in their case study on a uniform strain field. Due to the strain transfer in the fiber optic sensor, these boundary conditions obviously cannot be verified (i.e., the strain in the core of the optical fiber can absolutely not be equal to zero at x=0 and x=L). Moreover, this is what can be observed in the measurement results presented in [19], which do not correspond well to the theoretical results, especially in the vicinity of the boundary conditions. In order to better match the calculated and measured strain profiles at x=0 and x=L, ref. [20] recently proposed considering a percentage of residual strain at the boundary conditions. However, it should be noted that this parameter is fixed arbitrarily without any theoretical basis.

In this paper, a strain transfer analysis on a five-layer model that is more representative of common optical cables available on the market is presented. This modeling considers the bonding condition for all the interfaces of a multi-layer system, but no assumptions are made on the shear modulus of the coating layers unlike the model proposed by Bassil. This results in a new expression for the strain lag parameter. In addition, a method for determining the strain profile of a distributed fiber optic sensor under any arbitrary strain distribution in the host material is proposed for the first time. It is important to underline that a new set of boundary conditions is used to obtain a general solution. From the latter, closed-form solutions of the strain transfer function can be deduced. In order to show, in practice, how the general solution is to be used, the calculations of closed-form for uniform and non-uniform strain fields are presented. Then, experimental measurements obtained with a high spatial resolution Optical Frequency Domain Reflectometer (OFDR) are confronted with the results calculated from the derived formulas in order to demonstrate the validity of the new boundary conditions and the precision of the method.

## 2. Analytical Modeling of the Strain Transfer

This section describes the geometrical model, the assumptions and the calculation of the governing equation for the strain transfer in distributed optical fiber sensors embedded into a host material from which a new expression of the strain-lag parameter is obtained. It will be also be shown that the obtained governing equation can also be used for surface-mounted optical fibers with another expression for the strain-lag parameter. Then, the importance of the strain-lag parameter for the characterization of the strain transfer is discussed.

### 2.1. Governing Equation

In distributed strain measurements, the sensing element is a single-monomode optical fiber similar to the ones used in telecommunication systems. Typically, the optical fiber is composed of a glass core (around 10 μm) surrounded by an optical cladding made of glass (with a lower refractive index than the core’s index) of 125 μm diameter. Since bare optical fiber is brittle, a protective coating with polymer is added. In general, single or dual acrylate coating, polyimide coating, Ormocer coating (the latest coating proposed in the market) and/or silicone coating (for applications requiring higher temperature performance) are used. According to the type of coating and its manufacturer, the external diameter of the optical fiber can vary from from 150 μm to 300 μm. These kinds of optical fibers are well adapted for surface-mounted applications, as shown in the study of Weisbrich [21] which compares different fiber coatings and adhesives on steel surfaces for distributed optical strain measurements. For embedded optical fibers in a host material (such as concrete), more robust sensors are used to prevent damage during the installation. The optical fibers are usually packaged with additional protective coatings. For these sensors, often called optical cables, the outer diameter can vary approximately from 0.9 μm to 5 mm. Thus, in order to cover the large variety of optical cables used in real applications, a five-layer model is chosen in this study. Note that this choice has an impact only on the strain-lag parameter as it will be discussed later in this paper.

**Figure 1:**
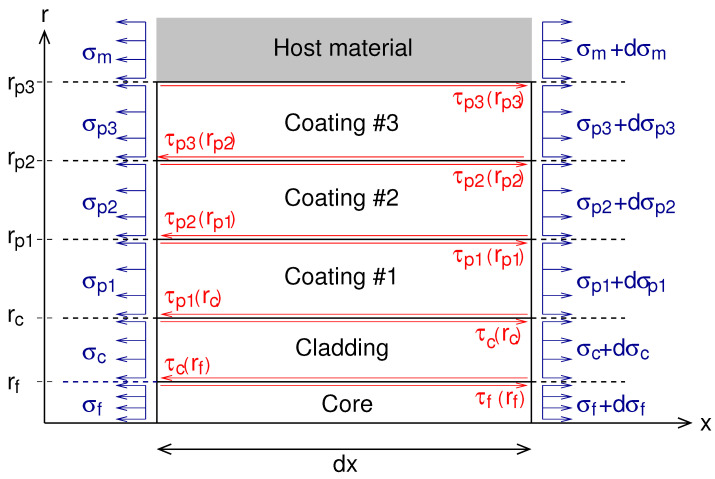
Schematic diagram showing the geometry of the optical fiber sensor embedded in a host material and the stresses used in the stress-strain analysis.

Figure 1 shows the geometry of the five-layer model with the different normal stresses and shear stresses that occur in an infinitesimal part of the optical cable when the host material is subjected to an arbitrary longitudinal stress. Due to symmetry, only half of the system is analyzed. The outer radii of different layers are denoted as $r_{f}$, $r_{c}$, $r_{p\;1}$, $r_{p\;2}$ and $r_{p\;3}$ for the radius of core, optical cladding, coating #1, coating #2 and coating #3, respectively. The longitudinal stress in the host material is transferred by shear stresses to different layers of the optical sensor up to the fiber’s core where the light of the laser of the optical interrogator propagates. The shear stresses τ and normal stresses σ in the fiber’s core, in the optical cladding, in the coating #1, in the coating #2 and in coating #3 are denoted as $\tau_{f}$, $\sigma_{f}$, $\tau_{c}$, $\sigma_{c}$, $\tau_{p\;1}$, $\sigma_{p\;1}$, $\tau_{p\;2}$, $\sigma_{p\;2}$ and $\tau_{p\;3}$, $\sigma_{p\;3}$ respectively. The assumptions used in the stress-strain transfer analysis proposed in this paper are presented as follows.

**Assumption** **1.** *The host material and all the materials that make up the optical fiber are supposed to be isotropic materials and have linear elastic behaviors.*

**Assumption** **2.** *The optical fiber cable deforms essentially under the effect of the tensile stress and the Poisson effect is considered negligible.*

**Assumption** **3.** *A perfect bonding at the interface between the optical fiber core and the optical fiber cladding is assumed.*

**Assumption** **4.** *The behavior of all the interfaces (with the exception of the interface between the optical fiber core and the optical fiber cladding) is considered to be imperfect and can be modeled as follows:*

(1) $$\tau_{i}=k_{i/j}\;\Delta u_{i/j}$$

*where $\tau_{i}$ is the shear stress in the layer i at the interface with the adjacent layer j and $\Delta u_{i/j}$ is the relative displacement between the two layers i and j. The coefficient $k_{i/j}$ represents the interface stiffness parameter. For $k_{i/j}=1$, the two adjacent layers i and j are perfectly bonded and, conversely, for $k_{i/j}=0$, they are fully debonded.*

**Assumption** **5.** *The strain gradients are expected to be of the same order for adjacent layers:*

(2) $$\frac{d\epsilon_{i}\left(x\right)}{dx}\approx \frac{d\epsilon_{j}\left(x\right)}{dx}$$

*where i and j refer to c, f, p1, p2 and p3, respectively, for the layers designated by core, optical cladding, coating #1, coating #2 and coating #3 in Figure 1.*

From the Assumptions 1 and 2, the relations between stress and strain in the different components of the modeled optical cable are provided by Hooke’s law:

(3a) $$\begin{array}{cc} \sigma_{i}= & \;E_{i}\;\epsilon_{i} \end{array}$$

(3b) $$\begin{array}{cc} \tau_{i}= & \;G_{i}\;\frac{\partial u_{i}}{\partial r} \end{array}$$

where $E_{i}$ and $G_{i}$ are the Young’s modulus and the shear modulus coefficients of the material of the layer *i*, respectively.

By applying Newton’s third law, the equations of equilibrium can be written as the following for each part of the optical cable shown in Figure 1.

$$\begin{array}{c} 0\leq r\leq r_{f}\;\; \end{array}$$

(4a) $$\begin{array}{cc} \; & \pi \;r^{2}\;\left(\sigma_{f}+d\sigma_{f}\right)-\pi \;r^{2}\;\sigma_{f}+2\pi \;r\int_{0}^{dx}\tau_{f}\left(\xi ,r\right)\;d\xi =0\;\\ \;r_{f}\leq r\leq r_{c} & \end{array}$$

(4b) $$\begin{array}{cc} \; & \pi \;\left(r^{2}-r_{f}^{2}\right)\left(\sigma_{c}+d\sigma_{c}\right)-\pi \;\left(r^{2}-r_{f}^{2}\right)\;\sigma_{c}+2\;\pi \;r\int_{0}^{dx}\tau_{c}\left(\xi ,r\right)\;d\xi -2\;\pi \;r_{f}\int_{0}^{dx}\tau_{f}\left(\xi ,r_{f}\right)\;d\xi =0\;\\ \;r_{c}\leq r\leq r_{p1} & \end{array}$$

(4c) $$\begin{array}{cc} \; & \pi \;\left(r^{2}-r_{c}^{2}\right)\left(\sigma_{p1}+d\sigma_{p1}\right)-\pi \;\left(r^{2}-r_{c}^{2}\right)\;\sigma_{p1}+2\;\pi \;r\int_{0}^{dx}\tau_{p1}\left(\xi ,r\right)\;d\xi -2\;\pi \;r_{c}\int_{0}^{dx}\tau_{c}\left(\xi ,r_{c}\right)\;d\xi =0\\ r_{p1}\leq r\leq r_{p2} & \end{array}$$

(4d) $$\begin{array}{cc} \; & \pi \;\left(r^{2}-r_{p1}^{2}\right)\left(\sigma_{p2}+d\sigma_{p2}\right)-\pi \;\left(r^{2}-r_{p1}^{2}\right)\;\sigma_{p2}+2\;\pi \;r\int_{0}^{dx}\tau_{p2}\left(\xi ,r\right)\;d\xi -2\;\pi \;r_{p1}\int_{0}^{dx}\tau_{p1}\left(\xi ,r_{p1}\right)\;d\xi =0\\ \;r_{p2}\leq r\leq r_{p3} & \end{array}$$

(4e) $$\begin{array}{cc} \; & \pi \;\left(r^{2}-r_{p2}^{2}\right)\left(\sigma_{p3}+d\sigma_{p3}\right)-\pi \;\left(r^{2}-r_{p2}^{2}\right)\;\sigma_{p3}+2\;\pi \;r\int_{0}^{dx}\tau_{p3}\left(\xi ,r\right)\;d\xi -2\;\pi \;r_{p2}\int_{0}^{dx}\tau_{p2}\left(\xi ,r_{p2}\right)\;d\xi =0 \end{array}$$

By assuming that the shear stresses $\tau_{c}\left(x\right)$, $\tau_{f}\left(x\right)$, $\tau_{p1}\left(x\right)$, $\tau_{p2}\left(x\right)$ and $\tau_{p3}\left(x\right)$ are constant along the micro-section of length dx, the previous equations can be rewritten as follows.

(5a) $$\begin{array}{cc} 0\leq r\leq r_{f}\; & \tau_{f}\left(x,r\right)=-\frac{r}{2}\;\frac{d\sigma_{f}\left(x\right)}{dx}\; \end{array}$$

(5b) $$\begin{array}{cc} r_{f}\leq r\leq r_{c}\; & \tau_{c}\left(x,r\right)=-\frac{r^{2}-r_{f}^{2}}{2\;r}\;\frac{d\sigma_{c}\left(x\right)}{dx}+\frac{r_{f}}{r}\;\tau_{f}\left(x,r_{f}\right)\; \end{array}$$

(5c) $$\begin{array}{cc} r_{c}\leq r\leq r_{p1}\; & \tau_{p1}\left(x,r\right)=-\frac{r^{2}-r_{c}^{2}}{2\;r}\;\frac{d\sigma_{p1}\left(x\right)}{dx}+\frac{r_{c}}{r}\;\tau_{c}\left(x,r_{c}\right)\; \end{array}$$

(5d) $$\begin{array}{cc} r_{p1}\leq \;r\leq r_{p2}\; & \tau_{p2}\left(x,r\right)=-\frac{r^{2}-r_{p1}^{2}}{2\;r}\;\frac{d\sigma_{p2}\left(x\right)}{dx}+\frac{r_{p1}}{r}\;\tau_{p1}\left(x,r_{p1}\right) \end{array}$$

(5e) $$\begin{array}{cc} r_{p2}\leq \;r\leq r_{p3}\; & \tau_{p3}\left(x,r\right)=-\frac{r^{2}-r_{p2}^{2}}{2\;r}\;\frac{d\sigma_{p3}\left(x\right)}{dx}+\frac{r_{p2}}{r}\;\tau_{p2}\left(x,r_{p2}\right) \end{array}$$

With Assumption 5 and Equation (3a), the new following relations can be obtained as follows.

(6a) $$\begin{array}{cc} 0\leq \;r\leq r_{f} & \\ \tau_{f}\left(x,r\right)= & -\frac{r}{2}\;\frac{d\sigma_{f}\left(x\right)}{dx} \end{array}$$

(6b) $$\begin{array}{cc} r_{f}\leq \;r\leq r_{c} & \\ \tau_{c}\left(x,r\right)= & -(\frac{r^{2}-r_{f}^{2}}{2\;r}\;\frac{E_{c}}{E_{f}}+\frac{r_{f}^{2}}{2\;r})\frac{d\sigma_{f}\left(x\right)}{dx} \end{array}$$

(6c) $$\begin{array}{cc} r_{c}\leq \;r\leq r_{p1} & \\ \tau_{p1}\left(x,r\right)= & -(\frac{r^{2}-r_{c}^{2}}{2\;r}\;\frac{E_{p1}}{E_{f}}+\frac{r_{c}^{2}-r_{f}^{2}}{2\;r}\;\frac{E_{c}}{E_{f}}+\frac{r_{f}^{2}}{2\;r})\frac{d\sigma_{f}\left(x\right)}{dx} \end{array}$$

(6d) $$\begin{array}{cc} r_{p1}\leq \;r\leq r_{p2} & \\ \tau_{p2}\left(x,r\right)= & -(\frac{r^{2}-r_{p1}^{2}}{2\;r}\;\frac{E_{p2}}{E_{f}}+\frac{r_{p1}^{2}-r_{c}^{2}}{2\;r}\;\frac{E_{p1}}{E_{f}}+\frac{r_{c}^{2}-r_{f}^{2}}{2\;r}\;\frac{E_{c}}{E_{f}}+\frac{r_{f}^{2}}{2\;r})\frac{d\sigma_{f}\left(x\right)}{dx} \end{array}$$

(6e) $$\begin{array}{cc} r_{p2}\leq \;r\leq r_{p3}\\ \tau_{p3}\left(x,r\right)= & -(\frac{r^{2}-r_{p2}^{2}}{2\;r}\;\frac{E_{p3}}{E_{f}}+\frac{r_{p2}^{2}-r_{p1}^{2}}{2\;r}\;\frac{E_{p2}}{E_{f}}+\frac{r_{p1}^{2}-r_{c}^{2}}{2\;r}\;\frac{E_{p1}}{E_{f}}+\frac{r_{c}^{2}-r_{f}^{2}}{2\;r}\;\frac{E_{c}}{E_{f}}+\frac{r_{f}^{2}}{2\;r})\frac{d\sigma_{f}\left(x\right)}{dx} \end{array}$$

According to Equation (3b), by integrating the shear stresses given by Equations (6b)–(6e), the relative displacement between the different layers of the system can be expressed as follows.

(7a) $$\begin{array}{cc} u_{c}\left(x,r_{f}\right)-u_{c}\left(x,r_{c}\right)= & -\frac{1}{G_{f}}\;[(\frac{r_{f}^{2}-r_{c}^{2}}{4}-\frac{r_{c}^{2}}{2}\;\ln(\frac{r_{f}}{r_{c}}))\frac{E_{f}}{E_{c}}+\frac{r_{c}^{2}}{2}\;\ln(\frac{r_{f}}{r_{c}})]\;\frac{d\sigma_{f}\left(x\right)}{dx}\\ u_{p\;1}\left(x,r_{p\;1}\right)-u_{p\;1}\left(x,r_{f}\right)= & -\frac{1}{G_{p\;1}}\;[(\frac{r_{p\;1}^{2}-r_{f}^{2}}{4}-\frac{r_{f}^{2}}{2}\;\ln(\frac{r_{p\;1}}{r_{f}}))\frac{E_{p\;1}}{E_{c}}+ \end{array}$$

(7b) $$\begin{array}{cc} \; & (\frac{r_{f}^{2}-r_{c}^{2}}{2}\;\frac{E_{f}}{E_{c}}+\frac{r_{c}^{2}}{2})\;\ln(\frac{r_{p\;1}}{r_{f}})]\;\frac{d\sigma_{f}\left(x\right)}{dx}\\ u_{p\;2}\left(x,r_{p\;2}\right)-u_{p\;2}\left(x,r_{p\;1}\right)= & -\frac{1}{G_{p\;2}}\;[(\frac{r_{p\;2}^{2}-r_{p\;1}^{2}}{4}-\frac{r_{p\;1}^{2}}{2}\;\ln(\frac{r_{p\;2}}{r_{p\;1}}))\frac{E_{p\;2}}{E_{c}}+ \end{array}$$

(7c) $$\begin{array}{cc} \; & (\frac{r_{p\;1}^{2}-r_{f}^{2}}{2}\;\frac{E_{p\;1}}{E_{c}}+\frac{r_{f}^{2}-r_{c}^{2}}{2}\;\frac{E_{f}}{E_{c}}+\frac{r_{c}^{2}}{2})\;\ln(\frac{r_{p\;2}}{r_{p\;1}})]\;\frac{d\sigma_{f}\left(x\right)}{dx}\\ u_{p\;3}\left(x,r_{p\;3}\right)-u_{p\;3}\left(x,r_{p\;2}\right)= & -\frac{1}{G_{p\;3}}\;[(\frac{r_{p\;3}^{2}-r_{p\;2}^{2}}{4}-\frac{r_{p\;2}^{2}}{2}\;\ln(\frac{r_{p\;3}}{r_{p\;2}}))\frac{E_{p\;3}}{E_{c}}+ \end{array}$$

(7d) $$\begin{array}{cc} \; & (\frac{r_{p\;2}^{2}-r_{p\;1}^{2}}{2}\;\frac{E_{p\;2}}{E_{c}}+\frac{r_{p\;1}^{2}-r_{f}^{2}}{2}\;\frac{E_{p\;1}}{E_{c}}+\frac{r_{f}^{2}-r_{c}^{2}}{2}\;\frac{E_{f}}{E_{c}}+\frac{r_{c}^{2}}{2})\;\ln(\frac{r_{p\;3}}{r_{p\;2}})]\;\frac{d\sigma_{f}\left(x\right)}{dx} \end{array}$$

With the Assumptions 3 and 4, the following equations can also be obtained.

(8a) $$\begin{array}{cc} u_{f}\left(x,r_{f}\right)-u_{c}\left(x,r_{f}\right)= & 0 \end{array}$$

(8b) $$\begin{array}{cc} u_{p\;1}\left(x,r_{c}\right)-u_{f}\left(x,r_{c}\right)= & \frac{\tau_{c}\left(x,r_{c}\right)}{k_{c}} \end{array}$$

(8c) $$\begin{array}{cc} u_{p\;2}\left(x,r_{p\;1}\right)-u_{p\;1}\left(x,r_{p\;1}\right)= & \frac{\tau_{p\;1}\left(x,r_{p\;1}\right)}{k_{p\;1/2}} \end{array}$$

(8d) $$\begin{array}{cc} u_{p\;3}\left(x,r_{p\;2}\right)-u_{p\;2}\left(x,r_{p\;2}\right)= & \frac{\tau_{p\;2}\left(x,r_{p\;2}\right)}{k_{p\;2/3}} \end{array}$$

(8e) $$\begin{array}{cc} u_{m}\left(x,r_{p\;3}\right)-u_{p\;3}\left(x,r_{p\;3}\right)= & \frac{\tau_{p\;3}\left(x,r_{p\;3}\right)}{k_{p\;3/m}} \end{array}$$

The expressions of $\tau_{c}\left(x,r_{c}\right)$, $\tau_{p\;1}\left(x,r_{p\;1}\right)$, $\tau_{p\;2}\left(x,r_{p\;2}\right)$ and $\tau_{p\;3}\left(x,r_{p\;3}\right)$ are given by the relations (6b)–(6e), respectively. Furthermore, $u_{m}\left(x,r_{p\;3}\right)$ can be expressed as follows.

(9) $$\begin{array}{cc} u_{m}\left(x,r_{p\;3}\right)= & u_{m}\left(x,r_{p\;3}\right)-u_{p\;3}\left(x,r_{p\;3}\right)+u_{p\;3}\left(x,r_{p\;3}\right)-u_{p\;3}\left(x,r_{p\;2}\right)\\ \; & +u_{p\;3}\left(x,r_{p\;2}\right)-u_{p\;2}\left(x,r_{p\;2}\right)+u_{p\;2}\left(x,r_{p\;2}\right)-u_{p\;2}\left(x,r_{p\;1}\right)\\ \; & +u_{p\;2}\left(x,r_{p\;1}\right)-u_{p\;1}\left(x,r_{p\;1}\right)+u_{p\;1}\left(x,r_{p\;1}\right)-u_{f}\left(x,r_{p\;1}\right)\\ \; & +u_{c}\left(x,r_{p\;1}\right)-u_{c}\left(x,r_{c}\right)+u_{f}\left(x,r_{c}\right)-u_{c}\left(x,r_{f}\right)\\ \; & +u_{c}\left(x,r_{f}\right)-u_{f}\left(x,r_{f}\right)+u_{f}\left(x,r_{f}\right) \end{array}$$

By using Equations (7a)–(7d) and (8a)–(8e), the relation (9) can be reduced to the following case:

(10) $$u_{m}\left(x,r_{p\;3}\right)=-\lambda \frac{d\epsilon_{f}\left(x\right)}{dx}+u_{f}\left(x,r_{f}\right)$$

where λ is given by the following.

(11) $$\begin{array}{cc} \lambda = & \frac{E_{f}}{k_{p\;3/m}}(\frac{r_{p3}^{2}-r_{p2}^{2}}{2\;r_{p3}}\;\frac{E_{p3}}{E_{f}}+\frac{r_{p2}^{2}-r_{p1}^{2}}{2\;r_{p3}}\;\frac{E_{p2}}{E_{f}}+\frac{r_{p1}^{2}-r_{c}^{2}}{2\;r_{p3}}\;\frac{E_{p1}}{E_{f}}+\frac{r_{c}^{2}-r_{f}^{2}}{2\;r_{p3}}\;\frac{E_{c}}{E_{f}}+\frac{r_{f}^{2}}{2\;r_{p3}})\\ \; & +\frac{E_{f}}{G_{p\;3}}\;[(\frac{r_{p\;3}^{2}-r_{p\;2}^{2}}{4}-\frac{r_{p\;2}^{2}}{2}\;\ln(\frac{r_{p\;3}}{r_{p\;2}}))\frac{E_{p\;3}}{E_{f}}+\\ \; & \left(\frac{r_{p\;2}^{2}-r_{p\;1}^{2}}{2}\;\frac{E_{p\;2}}{E_{f}}+\frac{r_{p\;1}^{2}-r_{c}^{2}}{2}\;\frac{E_{p\;1}}{E_{f}}+\frac{r_{c}^{2}-r_{f}^{2}}{2}\;\frac{E_{c}}{E_{f}}+\frac{r_{f}^{2}}{2})\;\ln(\frac{r_{p\;3}}{r_{p\;2}})\right]\\ \; & +\frac{E_{f}}{k_{p\;2/3}}(\frac{r_{p2}^{2}-r_{p1}^{2}}{2\;r_{p2}}\;\frac{E_{p2}}{E_{f}}+\frac{r_{p1}^{2}-r_{c}^{2}}{2\;r_{p2}}\;\frac{E_{p1}}{E_{f}}+\frac{r_{c}^{2}-r_{f}^{2}}{2\;r_{p2}}\;\frac{E_{c}}{E_{f}}+\frac{r_{f}^{2}}{2\;r_{p2}})\\ \; & +\frac{E_{f}}{G_{p\;2}}\;[(\frac{r_{p\;2}^{2}-r_{p\;1}^{2}}{4}-\frac{r_{p\;1}^{2}}{2}\;\ln(\frac{r_{p\;2}}{r_{p\;1}}))\frac{E_{p\;2}}{E_{f}}+\\ \; & \left(\frac{r_{p\;1}^{2}-r_{c}^{2}}{2}\;\frac{E_{p\;1}}{E_{f}}+\frac{r_{c}^{2}-r_{f}^{2}}{2}\;\frac{E_{c}}{E_{f}}+\frac{r_{f}^{2}}{2})\;\ln(\frac{r_{p\;2}}{r_{p\;1}})\right]\\ \; & +\frac{E_{f}}{k_{p\;1/2}}(\frac{r_{p\;1}^{2}-r_{c}^{2}}{2\;r_{p\;1}}\;\frac{E_{p1}}{E_{f}}+\frac{r_{c}^{2}-r_{f}^{2}}{2\;r_{p\;1}}\;\frac{E_{c}}{E_{f}}+\frac{r_{f}^{2}}{2\;r_{p\;1}})\\ \; & +\frac{E_{f}}{G_{p\;1}}\;[(\frac{r_{p\;1}^{2}-r_{c}^{2}}{4}-\frac{r_{c}^{2}}{2}\;\ln(\frac{r_{p\;1}}{r_{c}}))\frac{E_{p\;1}}{E_{f}}+(\frac{r_{c}^{2}-r_{f}^{2}}{2}\;\frac{E_{c}}{E_{f}}+\frac{r_{f}^{2}}{2})\;\ln(\frac{r_{p\;1}}{r_{c}})]\\ \; & +\frac{E_{f}}{k_{c}}(\frac{r_{c}^{2}-r_{f}^{2}}{2\;r_{c}}\;\frac{E_{c}}{E_{f}}+\frac{r_{f}^{2}}{2\;r_{c}})\\ \; & +\frac{E_{f}}{G_{c}}\;[(\frac{r_{c}^{2}-r_{f}^{2}}{4}-\frac{r_{f}^{2}}{2}\;\ln(\frac{r_{c}}{r_{f}}))\frac{E_{c}}{E_{f}}+\frac{r_{f}^{2}}{2}\;\ln(\frac{r_{c}}{r_{f}})] \end{array}$$

By calculating the derivative of Equation (10), the strain in the core of the optical fiber sensor $\epsilon_{f}\left(x\right)$ can be linked to the strain in the host material $\epsilon_{m}\left(x\right)$ by the following expression:

(12) $$\frac{d^{2}\epsilon_{f}\left(x\right)}{dx^{2}}-\beta^{2}\;\epsilon_{f}\left(x\right)=-\beta^{2}\;\epsilon_{m}\left(x\right)$$

where the coefficient β is defined as the strain-lag parameter given by the following.

(13) $$\beta^{2}=\frac{1}{\lambda }$$

The coefficient β depends on the geometry of the optical fiber sensor, on the mechanical properties of the materials of the different layers and on the interface stiffness parameters between the layers. Note that the methodology used to establish Equation (12) can be applied to optical fiber sensors having more or fewer layers than the case study presented in this paper. For instance, the strain-lag parameter is given for a three-layer and four-layer system in Appendix A.

### 2.2. Discussion on the Governing Equation on the Strain-Lag Parameter

The strain transfer from the host material to the optical fiber core is, thus, given by the governing Equation (12) that depends on the strain-lag parameter β. This key parameter is an intrinsic characteristic of all fiber optic sensors. According to the value of β, the strain transfer can be significantly different from one sensor to another. To provide a better idea of the importance of this parameter, a three-layer system (a core, an optical cladding and a coating) with perfect bonding conditions at the interface is considered for the sake of simplicity and two optical fibers made with different coatings, acrylate and polyimide, are compared. The respective strain-lag parameters can be calculated from Equation (A2) with the optical fiber’s dimensions and material properties given in Table 1. The calculation produces two very different values: 90 m${}^{-1}$ and 6096 m${}^{-1}$ for the optical fibers with an acrylate coating and a polyimide coating, respectively. This result means that an optical fiber with a polyimide coating has better strain transfer than an optical fiber with an acrylate coating. More generally, the higher the strain-lag value, the shorter the distance is for a complete strain transfer from the host material to the core of the fiber sensor. This effect will be illustrated in the section presenting different strain profiles obtained for different kinds of strain distribution for $\epsilon_{m}\left(x\right)$. Moreover, it is important to point out that the values of the strain-lag determined in the example above correspond to an idealized case study, i.e., without taking into account the interface stiffness between all the interfaces of the system.

In practice, the strain-lag values will be lower than those obtained in the above example. To give a better idea of the impact of the stiffness of the interfaces, only the interface stiffness between the optical fiber and the host material is considered in addition to the above example. A value of $10^{3}$ GPa/m (chosen in the middle of the range of the values used in [16] for the finite element simulations of pull-out test on an optical cable embedded inside a concrete specimen) is taken for $k_{p\;1/m}$ in Equation (A2). By taking the interface stiffness between the optical fiber and the host material into account, the values of the strain-lag parameter changes from 90 m${}^{-1}$ to 87 m${}^{-1}$ and from 6096 m${}^{-1}$ to 235 m${}^{-1}$ for the optical fibers with an acrylate coating and with a polyimide coating, respectively. This result shows that the strain-lag parameter does not only depend on the mechanical properties of the optical fiber’s layers but also on its interaction with the host material. Some manufacturers have proposed fiber optic sensors with rough surface in order to increase their bonding with the host material.

In this paper, only the case of a fiber optic sensor embedded into the host material has been considered. However, other installation configurations exist. For steel specimens, they are generally glued directly on surfaces with adhesives. For concrete specimens, fiber optic sensors are often introduced inside a surface groove filled with epoxy adhesive. In these surface-mounted configurations, the adhesive stiffness has an important impact on strain transfer as shown by the studies comparing several types of adhesives in [23] with an optical fiber attached on the concrete specimen subjected to a three-point loading test. Moreover, it is worth noting that the fiber optic sensor placed in a groove can be considered as embedded in the adhesive. In a coarse way, the model presented in the first part of this paper can be used to estimate the strain transfer for the surface-mounted configuration in a groove by adding an additional layer to take the adhesive into account. For example, the strain-lag parameter can be compared with a four-layer model (Equation (A3)) for a polyimide fiber embedded in a groove with several adhesives having different Young’s and shear modulus. For instance, values of 10 MPa and 1000 MPa are chosen for the Young’s modulus and 3.8 MPa and 380 MPa for the shear modulus. According to the values given in Table 1 for the polyimide fiber and by considering an outer diameter of the second layer (adhesive layer) equal to 500 μm and an interface stiffness between the adhesive and the host material equal to $10^{3}$ GPa/m, the strain-lag parameter can vary from 142 m${}^{-1}$ with the soft adhesive to 368 m${}^{-1}$ with the stiff adhesive. This result shows that the distance for a complete strain transfer from the host material to the core of the fiber sensor is longer for soft adhesives (such as silicone) than for stiff adhesive (such as epoxy) in accordance with the results of the study [22] in which finite element simulations have been compared to experimental results obtained with an optical fiber sealed in a groove at the surface of concrete specimens subjected to compressive tests.

For surface-mounted optical fiber sensors without grooves, several analytical models are proposed in the literature [20,24,25]. Similar to the models for an embedded optical fiber sensor, they are based on equilibrium equations that assume the following: All materials have a linear elastic behavior, all the interfaces are supposed to be perfectly bonded and the strain is transferred from the host material to the fiber sensor only by shear. Only the shear stress at the interface between the coating and the adhesive is expressed differently in order to take the geometry of the adhesive layer (the height of the adhesive layer varies in the direction transverse to the optical fiber) into account. Consequently, the governing equation for the strain transfer is the same as Equation (12), established for embedded optical fiber sensors. Only the expression of the strain lag parameter is different. Thus, it is obviously the key parameter for describing the strain transfer in an optical fiber sensors, regardless of whether they are surface-mounted or embedded in a host material. Furthermore, it is important to point out that the strain lag parameter depends not only on the geometrical and mechanical properties of the optical fiber but also on the installation conditions that can differ from one application to another. For surface-mounted sensors, the quality of adhesive, the surface preparation, the operator’s skill, etc., can affect the stiffness of the interface between the optical fiber sensor and the substrate and consequently the strain-lag parameter. For embedded configurations, the fluidity and cohesiveness of the concrete, etc. can change the strain-lag parameter as shown in [18]. In addition, for complex structures of optical cables, the mechanical properties of the different components are often unknown (not given in the datasheet and sometimes even more unknown by the manufacturer itself) and laboratory tests must be performed [16]. Consequently, in practice, the strain-lag parameter is difficult to determine. However, it should be noted that the method used in [17] for the monitoring of concrete crack-openings allows measuring the strain lag parameter indirectly through the strain transfer model.

## 3. Generalized Solution for Determining the Strain Profile in an Optical Fiber under Any Arbitrary Strain Distribution

In the previous section, it was shown that the strain transfer governing equation in distributed optical fiber sensors, for both surface-mounted and embedded configurations, corresponds to the differential Equation (12). In this section, a general solution for determining strain profile in optical fiber $\epsilon_{f}\left(x\right)$ under any arbitrary strain distribution $\epsilon_{m}\left(x\right)$ (at the level of host material) is detailed, and two case studies are presented. It should be emphasized that the originality of our solution lies in the introduction of new boundary conditions. In our point of view, the ones used in [19] or [20] are not very adequate as already mentioned in the introduction.

### 3.1. Presentation of the Generalized Solution

Before introducing the new boundary conditions, it is important to mention that the general solution of the differential Equation (12) is given by the sum of the solution corresponding to the homogeneous equation and the particular solution $\epsilon_{f}^{p}\left(x\right)$ of the non-homogeneous equation:

(14) $$\epsilon_{f}\left(x\right)=A\;\exp\left(\beta \;x\right)+B\;\exp\left(-\beta \;x\right)+\epsilon_{f}^{p}\left(x\right)$$

where *A* and *B* are unknown constants that can be determined by the boundary conditions. The particular solution $\epsilon_{f}^{p}\left(x\right)$ can be expressed as follows(see Appendix B for the demonstration):

(15) $$\epsilon_{f}^{p}\left(x\right)=-\beta \int_{0}^{x}\epsilon_{m}\left(x^{\prime }\right)\;\sinh\left(\beta (x-x^{\prime })\right)\;dx^{\prime }$$

where $\epsilon_{m}\left(x\right)$ is the strain profile at the surface of the substrate where the optical fiber is fixed.

**Figure 2:**
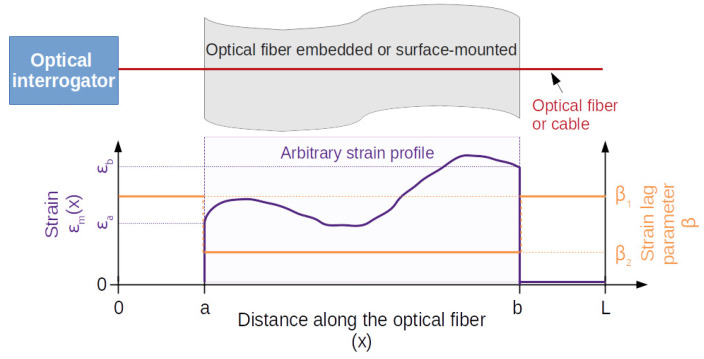
Arbitrary strain profile $\epsilon_{m}\left(x\right)$ between the abscissas *a* and *b* along the optical fiber of length *L*.

In order to obtain a general solution of Equation (12), the following boundary conditions are settled. As shown in Figure 2, an optical fiber sensor of a length *L* is considered. This part is localized between the abscissas noted *a* and *b* in Figure 2. The others two parts between the abscissas x=0 and x=a and between x=b and x=L are used to connect an optical interrogator and to make an optical termination. For these parts, the optical fiber is considered free. Consequently, $\epsilon_{m}\left(x\right)$ is assumed to be null if 0≤x<a and b<x≤L. On the other hand, no assumption is imposed on the shape of $\epsilon_{m}\left(x\right)$ between the abscissas x=a and x=b. This leads to the following boundaries.

$$\left\{ \begin{array}{cc} \epsilon_{f}\left(0\right)=0 & \text{ (16a)}\\ \epsilon_{f}\left(L\right)=0 & \text{ (16b)} \end{array}\right.$$

Note that the abscissas x=a and x=b cannot be equal to x=0 and x=L, respectively, for physical reasons (ends of the optical fiber), and the strain profile at the surface of the substrate, $\epsilon_{m}\left(x\right)$, at the abscissas x=a and x=b are $\epsilon_{m}\left(a\right)=\epsilon_{a}$ and $\epsilon_{m}\left(b\right)=\epsilon_{b}$, respectively, where $\epsilon_{a}$ and $\epsilon_{b}$ can take on any arbitrary values.

Since the strain measured by the optical fiber corresponds to a continuous and differentiable function at the abscissas x=a and x=b, the following conditions must also be verified.

(17a) $$\begin{array}{c} {\frac{d\epsilon_{f\;1}\left(x\right)}{dx}|}_{x=a}={\frac{d\epsilon_{f\;2}\left(x\right)}{dx}|}_{x=a} \end{array}$$

(17b) $$\begin{array}{c} {\frac{d\epsilon_{f\;2}\left(x\right)}{dx}|}_{x=b}={\frac{d\epsilon_{f\;3}\left(x\right)}{dx}|}_{x=b} \end{array}$$

**Figure 3:**
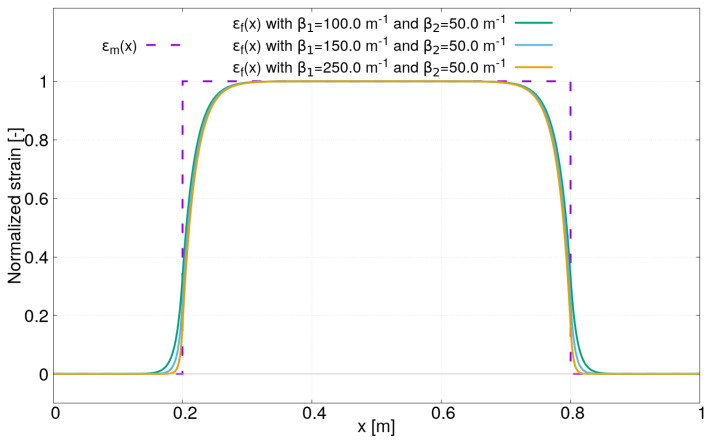
Strain profiles $\epsilon_{f}\left(x\right)$ under uniform field for different strain lag paramaters $\beta_{1}=100,150,250$ m${}^{-1}$ and $\beta_{2}=50$ m${}^{-1}$.

Moreover, since only a part of the optical fiber is used as a sensor, the strain lag parameter β is also assumed to be different between the abscissas x=a and x=b and those between x=0 and x=a and between x=b and x=L. For instance, for an optical fiber glued between x=a and x=b, the corresponding strain lag parameter should be lower (due to the layer of the adhesive) than the one corresponding to the part of the optical fiber between x=0 and x=a and between x=b and x=L (without the layer of the adhesive), as discussed in the previous section. As shown in the Figure 3, the strain lag parameter β is then defined by the following:

$$\left\{ \begin{array}{cc} \beta =\beta_{1}\;\mathrm{if}\;0\leq x<a\;\mathrm{or}\;b<x\leq L & \text{ (18a)}\\ \beta =\beta_{2}\;\mathrm{if}\;a\leq x\leq b & \text{ (18b)} \end{array}\right.$$

where $\beta_{1}$ and $\beta_{2}$ can take on any arbitrary values. From the statements on the strain profile $\epsilon_{m}\left(x\right)$ and the strain lag parameters $\beta_{1}$ and $\beta_{2}$, the particular solution $\epsilon_{f}^{p}\left(x\right)$ must, therefore, be determined by piecewise functions. Indeed, three cases must be taken into account according to the values of *x*.

✧ Case: 0≤x≤a

From Equation (15), we immediately obtain the following:

(19) $$\epsilon_{f\;1}^{p}\left(x\right)=0$$

and, consequently, the form of the general solution can be expressed as follows:

(20) $$\epsilon_{f\;1}\left(x\right)=A_{1}\;\exp\left(\beta_{1}\;x\right)+B_{1}\;\exp\left(-\beta_{1}\;x\right)$$

where $A_{1}$ and $B_{1}$ are constants.

✧ Case: a≤x≤b

From Equation (15), the particular solution is written as:

(21) $$\epsilon_{f\;2}^{p}\left(x\right)=-\beta_{2}\int_{a}^{x}\epsilon_{m}\left(x^{\prime }\right)\;\sinh\left(\beta_{2}\left(x-x^{\prime }\right)\right)\;dx^{\prime }$$

and the general solution is expressed by:

(22) $$\epsilon_{f\;2}\left(x\right)=A_{2}\;\exp\left(\beta_{2}\;x\right)+B_{2}\;\exp\left(-\beta_{2}\;x\right)+\epsilon_{f\;2}^{p}\left(x\right)$$

where $A_{2}$ and $B_{2}$ are constants.

✧ Case: b≤x≤L

According to Equation (15), the particular solution is as follows:

(23) $$\epsilon_{f\;3}^{p}\left(x\right)=-\beta_{1}\int_{a}^{b}\epsilon_{m}\left(x^{\prime }\right)\;\sinh\left(\beta_{1}\left(x-x^{\prime }\right)\right)\;dx^{\prime }$$

and, consequently, the form of the general solution can be written as the following:

(24) $$\epsilon_{f\;3}\left(x\right)=A_{3}\;\exp\left(\beta_{1}\;x\right)+B_{3}\;\exp\left(-\beta_{1}\;x\right)+\epsilon_{f\;3}^{p}\left(x\right)$$

where $A_{3}$ and $B_{3}$ are constants.

By using the boundary conditions (16a) to (16b) and the conditions (17a) to (17b), a set of equations can be obtained from which the constants $A_{1}$, $B_{1}$, $A_{2}$, $B_{2}$, $A_{3}$ and $B_{3}$ can be determined. Indeed, this set of equations can be rewritten in the following matrix form:

(25) $$\left[\begin{array}{cccc} 2\;\sinh(\beta_{1}\;a) & -\exp(\beta_{2}\;a) & -\exp(-\beta_{2}\;a) & 0\\ 2\;\cosh(\beta_{1}\;a) & -\exp(\beta_{2}\;a) & \exp(-\beta_{2}\;a) & 0\\ 0 & \exp(\beta_{2}\;b) & \exp(-\beta_{2}\;b) & -(\exp\left(\beta_{1}\;b\right)-\exp\left(\beta_{1}\left(2\;L-b\right)\right))\\ 0 & \exp(\beta_{2}\;b) & -\exp(-\beta_{2}\;b) & -\beta 1\;(\exp\left(\beta_{1}\;b\right)+\exp\left(\beta_{1}\left(2\;L-b\right)\right)) \end{array}\right]\;\left[\begin{array}{c} A_{1}\\ A_{2}\\ B_{2}\\ A_{3} \end{array}\right]=\left[\begin{array}{c} R_{1}\\ R_{2}\\ R_{3}\\ R_{4} \end{array}\right]$$

where the coefficients $R_{1}$, $R_{2}$, $R_{3}$ and $R_{4}$ can be expressed as the following.

$$\left\{ \begin{array}{cc} R_{1}\;=\;0 & \text{ (26a)}\\ R_{2}\;=\;0 & \text{ (26b)}\\ R_{3}\;=\;\epsilon_{f3}^{p}(b)\;-\;\epsilon_{f2}^{p}(b)\;-\;\epsilon_{f2}^{p}(L)\exp(\beta_{1}(L\;-\;b)) & \text{ (26c)}\\ R_{4}\;=\;{\frac{d\epsilon_{f3}^{p}\left(x\right)}{dx}|}_{x=b}\;-\;{\frac{d\epsilon_{f2}^{p}\left(x\right)}{dx}|}_{x=b}\;+\;\beta_{1}\epsilon_{f3}^{p}(L)\exp(\beta_{1}(L\;-\;b)) & \text{ (26d)} \end{array}\right.$$

By applying Cramer’s rule, the parameters $A_{1}$, $A_{2}$, $B_{2}$ and $A_{3}$ can be determined. Note that the constants $B_{1}$ and $B_{3}$ depends on the others parameters. In Appendix C, the reader can find a detailed presentation of the equations resulting in the matrix Equation (25) and the expressions of the parameters $A_{1}$, $A_{2}$, $B_{2}$ and $A_{3}$.

As a result, the general solution of Equation (12) can be then expressed as the following:

(27) $$\epsilon_{f}\left(x\right)=\left\{\begin{array}{ccc} \epsilon_{f\;1}\left(x\right) & \; & 0\leq x\leq a\\ \epsilon_{f\;2}\left(x\right) & \; & a\leq x\leq b\\ \epsilon_{f\;3}\left(x\right) & \; & b\leq x\leq L \end{array}\right.$$

where the following is the case:

(28a) $$\begin{array}{cc} \epsilon_{f\;1}\left(x\right)= & \frac{\beta_{2}^{2}}{\alpha }\{\left(\beta_{1}+\beta_{2}\right)\;\int_{a}^{b}\epsilon_{m}\left(x^{\prime }\right)[\exp(-\beta_{2}\;\left(2\;b-a-x^{\prime }\right)-\beta_{1}\;\left(x-2\;b+a+2\;L\right))\\ \; & \;-\exp\left(\beta_{2}\;\left(a-x^{\prime }\right)-\beta_{1}\;\left(x+a\right)\right)-\exp\left(-\beta_{2}\;\left(2\;b-a-x^{\prime }\right)+\beta_{1}\;\left(x+2\;b-a-2\;L\right)\right)\\ \; & \;+\exp\left(\beta_{2}\;\left(a-x^{\prime }\right)+\beta_{1}\;\left(x-a\right))\right]dx^{\prime }\\ \; & \;+\left(\beta_{2}-\beta_{1}\right)\;\int_{a}^{b}\epsilon_{m}\left(x^{\prime }\right)\left[\exp(\beta_{2}\;\left(a-x^{\prime }\right)-\beta_{1}\;\left(x-2\;b+a+2\;L\right))\right.\\ \; & \;+\exp\left(\beta_{1}\;\left(x-a\right)-\beta_{2}\;\left(2\;b-a-x^{\prime }\right)\right)-\exp\left(-\beta_{1}\;\left(x+a\right)-\beta_{2}\;\left(2\;b-a-x^{\prime }\right)\right)\\ \; & \;-\exp\left(\beta_{2}\;\left(a-x^{\prime }\right)+\beta_{1}\;\left(x+2\;b-a-2\;L\right)\right)]dx^{\prime }\} \end{array}$$

(28b) $$\begin{array}{cc} \epsilon_{f\;2}\left(x\right)= & \frac{\beta_{2}}{\alpha }\{\left(\frac{\beta_{1}^{2}+\beta_{2}^{2}}{2}\right)\;\int_{a}^{b}\epsilon_{m}\left(x^{\prime }\right)[\exp\left(-\beta_{2}\;\left(x-2\;a+2\;b-x^{\prime }\right)\right)-\exp\left(-\beta_{2}\;\left(x-2\;a+x^{\prime }\right)-2\;\beta_{1}\;a\right)\\ \; & \;+\exp\left(-\beta_{2}\;\left(x-2\;a+2\;b-x^{\prime }\right)-2\;\beta_{1}\;\left(L-b+a\right)\right)-\exp\left(\beta_{2}\;\left(x-2\;b+x^{\prime }\right)-2\;\beta_{1}\;\left(L-b\right)\right).\\ \; & \;-\exp\left(\beta_{2}\;\left(x-2\;b+x^{\prime }\right)-2\;\beta_{1}\;a\right)-\exp\left(-\beta_{2}\;\left(x-2\;a+x^{\prime }\right)-2\;\beta_{1}\;\left(L-b\right)\right)]dx^{\prime }\\ \; & \;+\left(\frac{\beta_{2}^{2}-\beta_{1}^{2}}{2}\right)\;\int_{a}^{b}\epsilon_{m}\left(x^{\prime }\right)[\exp\left(-\beta_{2}\;\left(x-2\;a+x^{\prime }\right)-2\;\beta_{1}\;\left(L-b+a\right)\right)+\exp\left(\beta_{2}\;\left(x-2\;b+x^{\prime }\right)\right)\\ \; & \;+\exp\left(\beta_{2}\;\left(x-2\;b+x^{\prime }\right)-2\;\beta_{1}\;\left(L-b+a\right)\right)-\exp\left(-\beta_{2}\;\left(x-2\;a+2\;b-x^{\prime }\right)-2\;\beta_{1}\;a\right).\\ \; & \;-\exp\left(-\beta_{2}\;\left(x-2\;a+2\;b-x^{\prime }\right)-2\;\beta_{1}\;\left(L-b\right)\right)+\exp\left(-\beta_{2}\;\left(x-2\;a+x^{\prime }\right))\right]dx^{\prime }\\ \; & \;+\beta_{1}\;\beta_{2}\;\int_{a}^{b}\epsilon_{m}\left(x^{\prime }\right)[\exp\left(-\beta_{2}\;\left(x-2\;a+x^{\prime }\right)-2\;\beta_{1}\;\left(L-b\right)\right)-\exp\left(-\beta_{2}\;\left(x-2\;a+2\;b-x^{\prime }\right)\right)\\ \; & \;+\exp\left(-\beta_{2}\;\left(x-2\;a+2\;b-x^{\prime }\right)-2\;\beta_{1}\;\left(L-b+a\right)\right)-\exp\left(\beta_{2}\;\left(x-2\;b+x^{\prime }\right)-2\;\beta_{1}\;\left(L-b\right)\right)\\ \; & \;+\exp\left(\beta_{2}\;\left(x-2\;b+x^{\prime }\right)-2\;\beta_{1}\;a\right)-\exp\left(-\beta_{2}\;\left(x-2\;a+x^{\prime }\right)-2\;\beta_{1}\;a\right))]dx^{\prime }\\ \; & \;+\left(\frac{\beta_{1}^{2}+\beta_{2}^{2}}{2}\right)\;\int_{a}^{x}\epsilon_{m}\left(x^{\prime }\right)[\exp\left(-\beta_{2}\;\left(x-x^{\prime }\right)\right)-\exp\left(-\beta_{2}\;\left(x+2\;b-2\;a-x^{\prime }\right)-2\;\beta_{1}\;\left(L-b+a\right)\right)\\ \; & \;+\exp\left(-\beta_{2}\;\left(2\;b-2\;a-x+x^{\prime }\right)-2\;\beta_{1}\;\left(L-b+a\right)\right)+\exp\left(-\beta_{2}\;\left(x-x^{\prime }\right)-2\;\beta_{1}\;\left(L-b+a\right)\right)\\ \; & \;-\exp\left(-\beta_{2}\;\left(x-2\;a+2\;b-x^{\prime }\right)\right)+\exp\left(-\beta_{2}\;\left(2\;b-2\;a-x+x^{\prime }\right))\right]dx^{\prime }\\ \; & \;+\left(\frac{\beta_{2}^{2}-\beta_{1}^{2}}{2}\right)\;\int_{a}^{x}\epsilon_{m}\left(x^{\prime }\right)[\exp\left(-\beta_{2}\;\left(2\;b-2\;a+x-x^{\prime }\right)-2\;\beta_{1}\;\left(L-b\right)\right)-\exp\left(-\beta_{2}\;\left(x-x^{\prime }\right)-2\;\beta_{1}\;a\right)\\ \; & \;+\exp\left(-\beta_{2}\;\left(2\;b-2\;a+x-x^{\prime }\right)-2\;\beta_{1}\;a\right)-\exp\left(-\beta_{2}\;\left(2\;b-2\;a-x+x^{\prime }\right)-2\;\beta_{1}\;a\right)\\ \; & \;-\exp\left(-\beta_{2}\;\left(2\;b-2\;a-x+x^{\prime }\right)-2\;\beta_{1}\;\left(L-b\right)\right)-\exp\left(-\beta_{2}\;\left(x-x^{\prime }\right)-2\;\beta_{1}\;\left(L-b\right)\right)]dx^{\prime }\\ \; & \;+\beta_{1}\;\beta_{2}\;\int_{a}^{x}\epsilon_{m}\left(x^{\prime }\right)[\exp\left(-\beta_{2}\;\left(x-x^{\prime }\right)\right)-\exp(-\beta_{2}\;\left(2\;b-2\;a+x-x^{\prime }\right)-2\;\beta_{1}\;\left(L-b+a\right).\\ \; & \;+\exp\left(-\beta_{2}\;\left(2\;b-2\;a-x+x^{\prime }\right)-2\;\beta_{1}\;\left(L-b+a\right)\right)-\exp\left(-\beta_{2}\;\left(2\;b-2\;a-x+x^{\prime }\right)\right)\\ \; & \;+\exp\left(-\beta_{2}\;\left(2\;b-2\;a+x-x^{\prime }\right)\right)-\exp\left(-\beta_{2}\;\left(x-x^{\prime }\right)-2\;\beta_{1}\;\left(L-b+a\right)\right))]dx^{\prime }\\ \; & \;+\left(\frac{\beta_{1}^{2}+\beta_{2}^{2}}{2}\right)\;\int_{x}^{b}\epsilon_{m}\left(x^{\prime }\right)[\exp\left(\beta_{2}\;\left(x-x^{\prime }\right)\right)+\exp\left(\beta_{2}\;\left(x-x^{\prime }\right)-2\;\beta_{1}\;\left(L-b+a\right)\right)]dx^{\prime }\\ \; & \;+\left(\frac{\beta_{1}^{2}-\beta_{2}^{2}}{2}\right)\;\int_{x}^{b}\epsilon_{m}\left(x^{\prime }\right)\left[\exp\left(\beta_{2}\;\left(x-x^{\prime }\right)-2\;\beta 1\;\left(L-b\right)\right)+\exp\left(\beta_{2}\;\left(x-x^{\prime }\right)-2\;\beta_{1}\;a\right)\right]dx^{\prime }\\ \; & \;+\left.\beta_{1}\;\beta_{2}\;\int_{x}^{b}\epsilon_{m}\left(x^{\prime }\right)[\exp\left(\beta_{2}\;\left(x-x^{\prime }\right)\right)-\exp\left(\beta_{2}\;\left(x-x^{\prime }\right)-2\;\beta_{1}\;\left(L-b+a\right))\right]dx^{\prime }\right\} \end{array}$$

(28c) $$\begin{array}{cc} \epsilon_{f\;3}\left(x\right)= & \frac{\beta_{2}^{2}}{\alpha }\{\left(\beta_{1}+\beta_{2}\right)\;\int_{a}^{b}\epsilon_{m}\left(x^{\prime }\right)\left[\exp(\beta_{1}\;\left(x-2\;a+b-2\;L\right)-\beta_{2}\;\left(b-2\;a+x^{\prime }\right)\right)\\ \; & \;-\exp\left(-\beta_{1}\;\left(x+2\;a-b\right)-\beta_{2}\;\left(b-2\;a+x^{\prime }\right)\right)+\exp\left(-\beta_{1}\;\left(x-b\right)-\beta_{2}\;\left(b-x^{\prime }\right)\right)\\ \; & \;-\exp\left(\beta_{1}\;\left(x+b-2\;L\right)-\beta_{2}\;\left(b-x^{\prime }\right)\right)]dx^{\prime }\\ \; & \;+\left(\beta_{2}-\beta_{1}\right)\;\int_{a}^{b}\epsilon_{m}\left(x^{\prime }\right)\left[\exp(-\beta_{1}\;\left(x-b\right)-\beta_{2}\;\left(b-2\;a+x^{\prime }\right))\right.\\ \; & \;-\exp\left(\beta_{1}\;\left(x+b-2\;L\right)-\beta_{2}\;\left(b-2\;a+x^{\prime }\right)\right)+\exp\left(\beta_{1}\;\left(x-2\;a+b-2\;L\right)-\beta_{2}\;\left(b-x^{\prime }\right)\right)\\ \; & \;-\exp\left(-\beta_{1}\;\left(x+2\;a-b\right)-\beta_{2}\;\left(b-x^{\prime }\right)\right)]dx^{\prime }\} \end{array}$$

The parameter α is given by:

(29) $$\begin{array}{cc} \alpha = & {\left(\beta_{1}-\beta_{2}\right)}^{2}\;\left(\exp\left(2\;\beta_{1}\;\left(b-a-L\right)\right)-\exp\left(-2\;\beta_{2}\;\left(b-a\right)\right)\right)\\ \; & +{\left(\beta_{1}+\beta_{2}\right)}^{2}\;\left(1-\exp(-2\;\beta_{2}\;\left(b-a\right)+2\;\beta_{1}\;\left(b-a-L\right))\right)\\ \; & +\left(\beta_{1}^{2}-\beta_{2}^{2}\right)\;\left(\exp\left(2\;\beta_{1}\;\left(b-L\right)\right)-\exp\left(-2\;\beta_{2}\;\left(b-a\right)-2\;\beta_{1}\;a\right)\right.\\ \; & \;\left.+\exp\left(-2\;\beta_{1}\;a\right)-\exp\left(-2\;\beta_{2}\;\left(b-a\right)+2\;\beta_{1}\;\left(b-L\right)\right)\right) \end{array}$$

It should be noted that the last Equations (28a)–(28c) depend only on $\epsilon_{m}\left(x\right)$. Consequently, the strain in the core of the optical fiber sensor can be determined for any arbitrary strain profile $\epsilon_{m}\left(x\right)$. It must be specified that analytical solutions can be found in some cases depending on the form of $\epsilon_{m}\left(x\right)$, as shown in the following section. For all others cases, numerical integration must be performed in order to obtain the strain profile $\epsilon_{f}\left(x\right)$ in the core of the optical fiber sensor. Note that the expressions of Equations (28a)–(28c) were written so as to avoid the computation of exponentials (or hyperbolic cosines and sines) with large positive integer exponents.

### 3.2. Applications

In this section, uniform and non-uniform strain fields for $\epsilon_{m}\left(x\right)$ are considered to show in detail how strain profiles $\epsilon_{f}\left(x\right)$ can be derived from Equations (28a)–(28c). The results will be then used to point out the impact of the strain-lag parameter value on the strain profiles measured $\epsilon_{f}\left(x\right)$ in comparison with the strain profile $\epsilon_{m}\left(x\right)$.

#### 3.2.1. Uniform Strain Field

First, the expression of the strain profile $\epsilon_{f}\left(x\right)$ is derived for a uniform strain field $\epsilon_{m}\left(x\right)$. As assumed in the previous section, $\epsilon_{m}\left(x\right)$ is defined between the abscissas x=a and x=b along the optical fiber of length *L* by the following:

(30) $$\begin{array}{ccc} \epsilon_{m}\left(x\right)=\epsilon_{m} & \; & a\leq x\leq b \end{array}$$

where $\epsilon_{m}$ is constant. As indicated by Equation (27), the strain profile $\epsilon_{f}\left(x\right)$ along the core of the optical fiber sensor under a uniform strain field can then be decomposed into three parts. From the integration of Equations (28a)–(28c), the following expression can be obtained for $\epsilon_{f\;1}\left(x\right)$, $\epsilon_{f\;2}\left(x\right)$ and $\epsilon_{f\;3}\left(x\right)$:

(31a) $$\begin{array}{cc} \epsilon_{f\;1}\left(x\right)=\epsilon_{m}\;\frac{\beta_{2}}{\alpha } & \{\left(\beta_{1}+\beta_{2}\right)\;\left(-\exp\left(-\beta_{1}\;\left(x+a\right)\right)-\exp\left(-\beta_{1}\;\left(x-2\;b+a+2\;L\right)-2\;\beta_{2}\;\left(b-a\right)\right)\right.\\ \; & \;\left.+\exp\left(\beta_{1}\;\left(x+a\right)\right)+\exp\left(\beta_{1}\;\left(x+2\;b-a-2,L\right)-2\;\beta_{2}\;\left(b-a\right)\right)\right)\\ \; & +\left(\beta_{2}-\beta_{1}\right)\;\left(\exp\left(-\beta_{1}\;\left(x-2\;b+a+2\;L\right)\right)+\exp\left(-\beta_{1}\;\left(x+a\right)-2\;\beta_{2}\;\left(b-a\right)\right)\right.\\ \; & \;\left.-\exp\left(\beta_{1}\;\left(x+2\;b-a-2\;L\right)\right)-\exp\left(\beta_{1}\;\left(x-a\right)-2\;\beta_{2}\;\left(b-a\right)\right)\right)\\ \; & +2\;\beta_{1}\;\left(\exp\left(-\beta_{1}\;\left(x+a\right)-\beta_{2}\;\left(b-a\right)\right)+\exp\left(-\beta_{1}\;\left(x-2\;b+a+2\;L\right)-\beta_{2}\;\left(b-a\right)\right)\right.\\ \; & \;-\exp\left(\beta_{1}\;\left(x-a\right)-\beta_{2}\;\left(b-a\right)\right)-\exp\left(-\beta_{1}\;\left(x+2\;b-a-2\;L\right)-\beta_{2}\;\left(b-a\right))\right)\} \end{array}$$

(31b) $$\begin{array}{cc} \epsilon_{f\;2}\left(x\right)=\epsilon_{m}\;\frac{\beta_{1}}{\alpha } & \{\left(\beta_{1}+\beta_{2}\right)\;\left(\exp\left(\beta_{2}\;\left(x-2\;b+a\right)-2\;\beta_{1}\;\left(L-b+a\right)\right)+\exp\left(-\beta_{2}\;\left(x-2\;a+b\right)-2\;\beta_{1}\;a\right)\right.\\ \; & \;-\exp\left(-\beta_{2}\;\left(x-2\;a+b\right)-2\;\beta_{1}\;\left(L-b+a\right)\right)-\exp\left(-\beta_{2}\;\left(x-a\right)\right)\\ \; & \;-\exp\left(\beta_{2}\;\left(x-b\right)-2\;\beta_{1}\;\left(L-b\right)\right)-\exp\left(-\beta_{2}\;\left(x-a\right)-2\;\beta_{1}\;a\right)\\ \; & \;\left.+\exp\left(\beta_{2}\;\left(x-2\;b+a\right)-2\;\beta_{1}\;\left(L-b\right)\right)-\exp\left(\beta_{2}\;\left(x-b\right)\right)\right)\\ \; & +\left(\beta_{2}-\beta_{1}\right)\;\left(\exp\left(-\beta_{2}\;\left(x-2\;a+b\right)-2\;\beta_{1}\;\left(L-b\right)\right)-\exp\left(\beta_{2}\;\left(x-b\right)-2\;\beta_{1}\;\left(L-b+a\right)\right)\right.\\ \; & \;-\exp\left(-\beta_{2}\;\left(x-a\right)-2\;\beta_{1}\;\left(L-b+a\right)\right)-\exp\left(-\beta_{2}\;\left(x-a\right)-2\;\beta_{1}\;\left(L-b\right)\right)\\ \; & \;+\exp\left(\beta_{2}\;\left(x-2\;b+a\right)-2\;\beta_{1}\;a\right)+\exp\left(-\beta_{2}\;\left(x-2\;a+b\right)\right)\\ \; & \;\left.+\exp\left(\beta_{2}\;\left(x-2\;b+a\right)\right)-\exp\left(\beta_{2}\;\left(x-b\right)-2\;\beta_{1}\;a\right)\right)\\ \; & +\frac{\left(\beta_{1}^{2}+\beta_{2}^{2}\right)}{\beta_{1}}\;\left(1-\exp\left(-2\;\beta_{2}\;\left(b-a\right)-2\;\beta_{1}\;\left(L-b+a\right)\right)+\exp\left(-2\;\beta_{1}\;\left(L-b+a\right)\right)\right.\\ \; & \;\left.-\exp(-2\;\beta_{2}\;\left(b-a\right))\right)\\ \; & +\frac{\left(\beta_{2}^{2}-\beta_{1}^{2}\right)}{\beta_{1}}\;\left(\exp\left(-2\;\beta_{2}\;\left(b-a\right)-2\;\beta_{1}\;\left(L-b\right)\right)+\exp\left(-2\;\beta_{2}\;\left(b-a\right)-2\;\beta_{1}\;a\right)\right.\\ \; & \;\left.+\exp\left(-2\;\beta_{1}\;\left(L-b\right)\right)-\exp\left(-2\;\beta_{1}\;a\right)\right)\\ \; & +\;2\;\beta_{2}\;\;\left(1-\exp\left(-2\;\beta_{2}\;\left(b-a\right)-2\;\beta_{1}\;\left(L-b+a\right)\right)-\exp\left(-2\;\beta_{1}\;\left(L-b+a\right)\right)\right.\\ \; & \;+\exp(-2\;\beta_{2}\;\left(b-a\right))\} \end{array}$$

(31c) $$\begin{array}{cc} \epsilon_{f\;3}\left(x\right)=\epsilon_{m}\;\frac{\beta_{2}}{\alpha } & \{\;\left(\beta_{1}+\beta_{2}\right)\;\left(\exp\left(-\beta_{1}\;\left(x+2\;a+b\right)-2\;\beta_{2}\;\left(b-a\right)\right)-\exp\left(\beta_{1}\;\left(x+b-2\;L\right)\right)\right.\\ \; & \;\left.-\exp\left(\beta_{1}\;\left(x-2\;a+b-2\;L\right)-2\;\beta_{2}\;\left(b-a\right)\right)-\exp\left(-\beta_{1}\;\left(x-b\right)\right)\right)\\ \; & +\left(\beta_{2}-\beta_{1}\right)\;\left(\exp\left(\beta_{1}\;\left(x+b-2\;L\right)-2\;\beta_{2}\;\left(b-a\right)\right)-\exp\left(-\beta_{1}\;\left(x-b\right)-2\;\beta_{2}\;\left(b-a\right)\right)\right.\\ \; & \;\left.+\exp\left(\beta_{1}\;\left(x-2\;a+b-2\;L\right)\right)-\exp\left(-\beta_{1}\;\left(x+2\;a-b\right)\right)\right)\\ \; & +2\;\beta_{1}\;\left(\exp\left(\beta_{1}\;\left(x-2\;a+b-2\;L\right)-\beta_{2}\;\left(b-a\right)\right)-\exp\left(-\beta_{1}\;\left(x+2\;a-b\right)-\beta_{2}\;\left(b-a\right)\right)\right.\\ \; & \;+\exp\left(\beta_{1}\;\left(x+b-2\;L\right)-\beta_{2}\;\left(b-a\right)\right)-\exp\left(-\beta_{1}\;\left(x-b\right)-\beta_{2}\;\left(b-a\right))\right)\} \end{array}$$

where the coefficient α is given by Equation (29).

It should be emphasized that these last expressions are only valid under a uniform strain field $\epsilon_{m}$ as defined by Equation (30). They are now used to investigate the effect of the strain lag parameters $\beta_{1}$ and $\beta_{2}$ on the strain profile $\epsilon_{f}\left(x\right)$. Three case studies are considered by varying a single parameter each time: $\beta_{1}$, $\beta_{2}$ or the distance between *a* and *b*. Figure 3 shows the first case study where only the strain lag parameter $\beta_{1}$ varies. Although the values $\beta_{1}$ are fixed arbitrarily, note that they remain in the range of values calculated in Section 2.2. Three strain profiles obtained for $\beta_{1}=100,150,250$ m${}^{-1}$ and $\beta_{2}=50$ m${}^{-1}$ are plotted in Figure 3. The other parameters for $\epsilon_{m}\left(x\right)$ in Equation (30) are fixed to the following: a=0.2 m, b=0.8 m and $\epsilon_{m}=1$ μm/m. Firstly, it can be observed that the strain profile $\epsilon_{f}\left(x\right)$ beyond the two ends (at x=a and x=b) where the uniform strain is applied along the sensor is not equal to zero due to the new boundary conditions proposed in this paper. Secondly, the strain lag parameter $\beta_{1}$ mainly has an influence on the shape of the strain profile $\epsilon_{f}\left(x\right)$ between the abscissas x=0 and x=a and between x=b and x=L, as expected. The higher the value of $\beta_{1}$, the faster $\epsilon_{f}\left(x\right)$ decreases to zero. Note also that the ratio $\beta_{1}/\beta_{2}$ has an influence on the level of strain in these areas.

**Figure 4:**
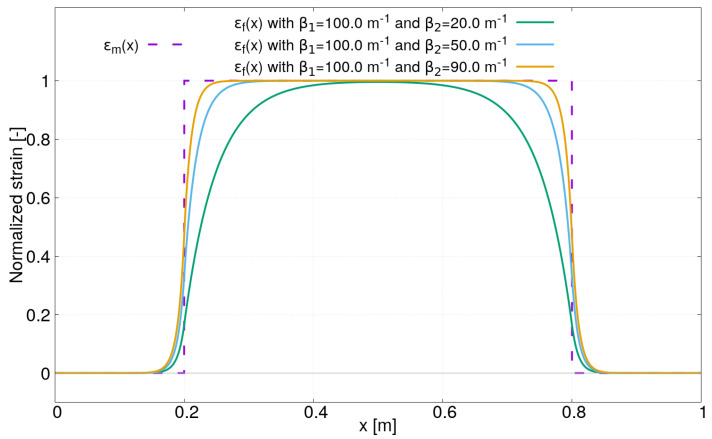
Strain profiles $\epsilon_{f}\left(x\right)$ under a uniform field for different strain lag parameters $\beta_{2}=20,50,90$ m${}^{-1}$ and $\beta_{1}=100$ m${}^{-1}$.

**Figure 5:**
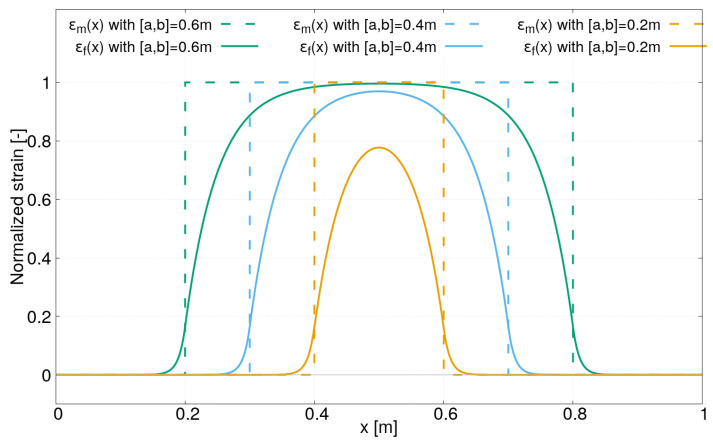
Strain profiles $\epsilon_{f}\left(x\right)$ (with $\beta_{1}=100\;m^{-1}$ and $\beta_{2}=20\;m^{-1}$) under uniform fields $\epsilon_{m}\left(x\right)$ of different lengths.

In the second case study, the strain lag parameter $\beta_{1}$ is fixed to 100 m${}^{-1}$ and three values for $\beta_{2}$ have been chosen: $\beta_{2}=20,50,90$ m${}^{-1}$. The strain profiles obtained for these latter values are plotted in Figure 4. It can be clearly observed that the distance over which the strain transfer occurs varies in function of $\beta_{2}$. As a result, the strain profile in the core of the optical sensor may be significantly different from the one along the optical sensor, especially for low values of $\beta_{2}$. We can also observe that after a certain distance, the strain in the core of the optical fiber $\epsilon_{f}\left(x\right)$ reaches the strain along the optical sensor $\epsilon_{m}\left(x\right)$. However, it also depends on $\epsilon_{m}\left(x\right)$, as shown in the Figure 5. The latter corresponds to the third case study where the two strain lag parameters $\beta_{1}$ and $\beta_{2}$ are fixed ($\beta_{1}=100\;m^{-1}$ and $\beta_{2}=20\;m^{-1}$), but the distance between the abscissas x=a et x=b varies from 0.2 m to 0.6 m. For a short distance between the abscissas x=a and x=b and for a low value of $\beta_{2}$, the strain transfer may be incomplete. As a result, the strain measured by the distributed optical fiber $\epsilon_{f}\left(x\right)$ may be strongly underestimated compared to the real strain in the host material $\epsilon_{m}\left(x\right)$.

#### 3.2.2. Non-Uniform Strain Field

**Figure 6:**
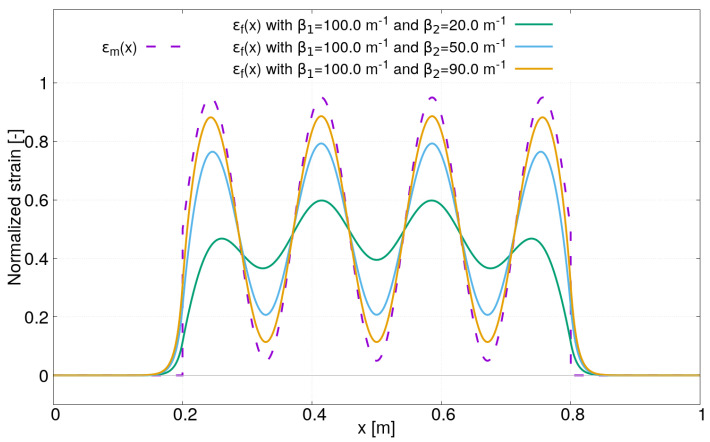
Strain distribution in the core of optical fiber sensor $\epsilon_{f}\left(x\right)$ induced by a sine strain field $\epsilon_{m}\left(x\right)$ in the host material.

In order to further demonstrate the importance of the strain lag parameters (especially $\beta_{2}$) on the strain profile $\epsilon_{f}\left(x\right)$, a non-uniform strain field is considered in this section. A complex strain profile $\epsilon_{m}\left(x\right)$ that exhibits high-spatial strain gradients has been chosen. It is expressed as follows:

(32) $$\epsilon_{m}\left(x\right)=\epsilon_{m}+2\;A\;\sin\left(\frac{x-a}{b-a}\;\left(2\;k+1\right)\;\pi \right)$$

where *k* is an integer. The shape of $\epsilon_{m}\left(x\right)$ is shown in Figure 6.

In order to determine the strain profile $\epsilon_{f}\left(x\right)$ in the core of the optical sensor that is induced by this non-uniform strain field, Equations (28a)–(28c) are used once again. Contrary to the previous case study with a uniform strain field, where analytical expressions have been derived, $\epsilon_{f}\left(x\right)$ was obtained for this case study by using a numerical calculation with Simpson’s rule (although an analytical expression can be derived with the strain profile $\epsilon_{m}\left(x\right)$ defined by Equation (32)). The following parameters have been used for $\epsilon_{m}\left(x\right)$ in Equation (32): a=0.2 m, b=0.8 m, $\epsilon_{m}=0.5$, A=0.225 and k=3. For the strain lag parameters, $\beta_{1}$ is fixed to 100 m${}^{-1}$ and three values for $\beta_{2}$ have been chosen: $\beta_{2}=20,50,90$ m${}^{-1}$. Figure 6 presents the strain profiles $\epsilon_{f}\left(x\right)$ obtained with these parameters. As expected, the strain lag parameter $\beta_{2}$ has an important effect on the strain profile $\epsilon_{f}\left(x\right)$ measured in the core of the optical fiber sensor. The lower the strain lag parameter $\beta_{2}$ is, the more the strain $\epsilon_{f}\left(x\right)$ is underestimated. Thus, for practical application with the presence of high-spatial strain gradients, it is of paramount importance to use fiber optic sensors characterized by a high strain lag parameter in order to avoid misinterpretation of distributed optical fiber sensor measurements.

## 4. Experimentation and Results

In order to validate the general solution (Equations (28a)–(28c)), experimental tests have been carried out. Firstly, the results obtained for a uniform strain field $\epsilon_{m}\left(x\right)$ will be presented and Equations (31a)–(31c), derived in Section 3.2.1, will be used to estimate the strain lag parameters $\beta_{1}$ and $\beta_{2}$. Then, the general solution is applied to a non-uniform strain field. The strain profiles calculated and measured will be then compared to show the efficiency of the method proposed in this paper.

### 4.1. Uniform Strain Field

#### 4.1.1. Description of the Experiment

**Figure 7:**
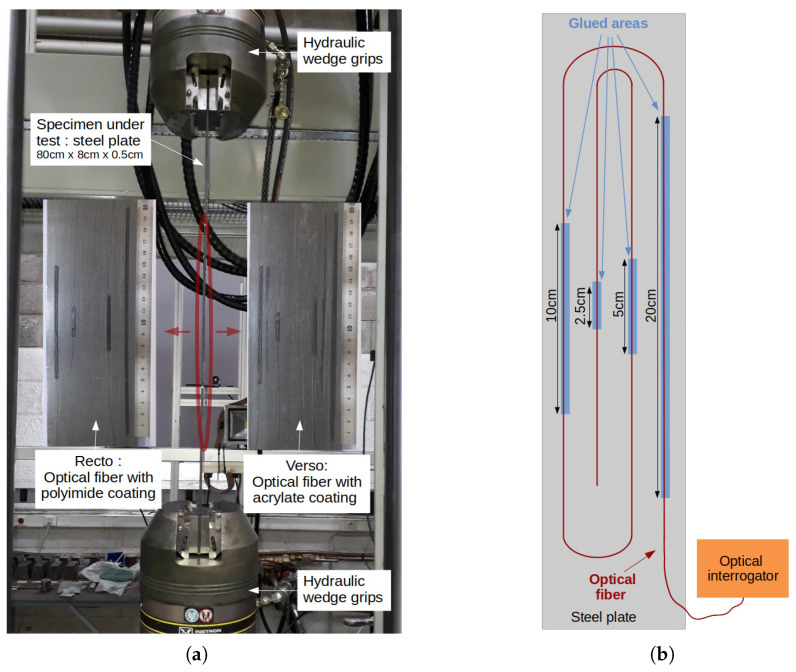
Experiment description of uniform strain field measurements. (**a**) Photography of the instrumented specimen under tensile test. (**b**) Schematic presentation of the specimen instrumentation.

A uniaxial tensile test on a steel plate (dimensions: 80 cm × 8 cm × 5 mm; Young’s Modulus: 200 MPa) has been performed to create a uniform strain field for $\epsilon_{m}\left(x\right)$. As shown in Figure 7a, a servohydraulic testing system applied a constant tensile force of 72 kN ($\sigma_{m}=180$ MPa) on the specimen during the measurements. The specimen has been instrumented on both sides by two monomode optical fibers possessing different coatings: polyimide (external diameter: 145 μm) and acrylate (external diameter: 250 μm). The two optical fibers have been attached on the specimen with a two-component epoxy adhesive (Araldite 2014-2) over four different lengths (20 cm, 10 cm, 5 cm and 2.5 cm), as shown in Figure 7b, in order to study the strain transfer effect as discussed in Section 3.2.1. The distributed strain measurements have been obtained with a high spatial resolution distributed optical fiber interrogator (0.65 mm gage pitch, 1.3 mm gage length, model: Luna ODISI-B). The operating principle of this system will not be described in this paper (the readers can refer to the following publications [26,27]). Nevertheless, it is important to remember that a strain profile is obtained from two optical measurements performed at different states. In this experiment, a reference measurement was set at null tensile force and the other at 72 kN, which corresponds to uniform strain of $\epsilon_{m}=$ 900 μm along the specimen between the wedge grips of the hydraulic testing system. Note also that the two optical fiber sensors have been interrogated one after the other. Consequently, the tensile tests have been repeated twice, and the force and displacement sensors have been used to control the repeatability of the tests. It was not possible to measure the two optical fibers at the same time due to the vibrations of the servohydraulic testing system inducing a cumulative noise on the strain measurement. Thus, a shorter length of optical fiber was used, which explains why the two optical fiber sensors have been interrogated one after the other.

#### 4.1.2. Results and Discussions

**Figure 8:**
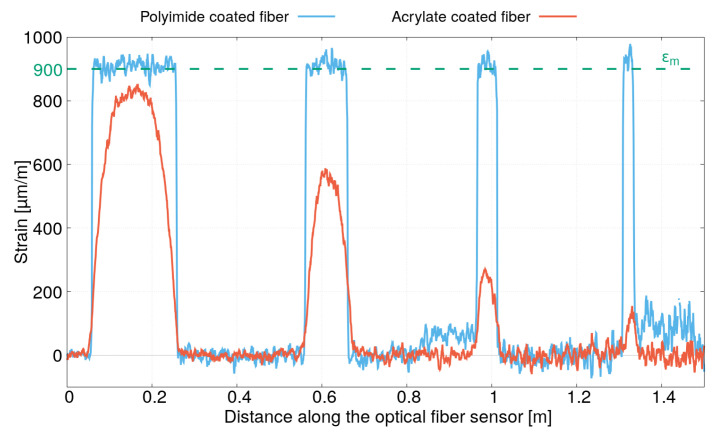
Strain profiles obtained for the sample instrumented by optical fibers with acrylate and polyimide coating under tensile test ($\sigma_{m}=180$ MPa).

Figure 8 presents the entire strain profile measured for the two optical fibers possessing polyimide and acrylate coatings. The four bonding lengths are clearly visible and the strain profiles for the two optical fibers are very different, as expected. Note also that the level of noise (due in large part to vibrations of the hydraulic testing system) increases over the length of the optical fiber. Moreover, two continuous noisy peaks emerge for polyimide-coated fiber between x=0.8 m and x=1 m and after x=1.3 m. They correspond to a small tension in the free portions of the optical fiber where small adhesive tapes are used to hold them to the steel plate (and to prevent them from being broken during handling). For the acrylate-coated fiber, the adhesive tapes were better placed preventing any tension during the tensile test. Note, however, that this instrumentation artifact has a very small influence on the strain profile measurements (less that 5% of the maximum strain), and it is not the cause of the differences between the strain profiles measured for both optical fibers having a polyimide and acrylate coating. Indeed, for the polyimide-coated fiber, the maximum strain value for each bonding length is equal to about 900 μm/m, which corresponds to the value of the strain in the steel plate imposed by the tensile test. For the acrylate-coated fiber, the maximum strain value is different for each bonding length. Shorter bonding lengths result in lower maximum strain values. This corresponds well to the observation deduced in Section 3.2.1 due to the strain transfer in the optical fiber sensor. The polyimide-coated fiber has a shorter strain transfer length than the one with an acrylate coating. The strain lag parameters $\beta_{1}$ and $\beta_{2}$, which drive the strain transfer mechanism of the two optical fiber sensors tested in this experiment, can be estimated by fitting the strain profiles corresponding to each of the four bonding lengths using Equations (31a)–(31c).

**Figure 9:**
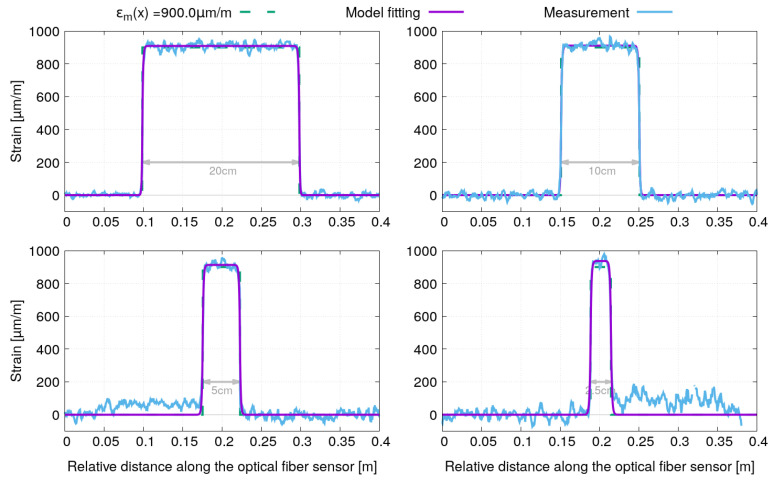
Fitting of the strain profiles obtained for the sample instrumented by optical fibers with polyimide coating under tensile test ($\sigma_{m}=180$ MPa).

**Figure 10:**
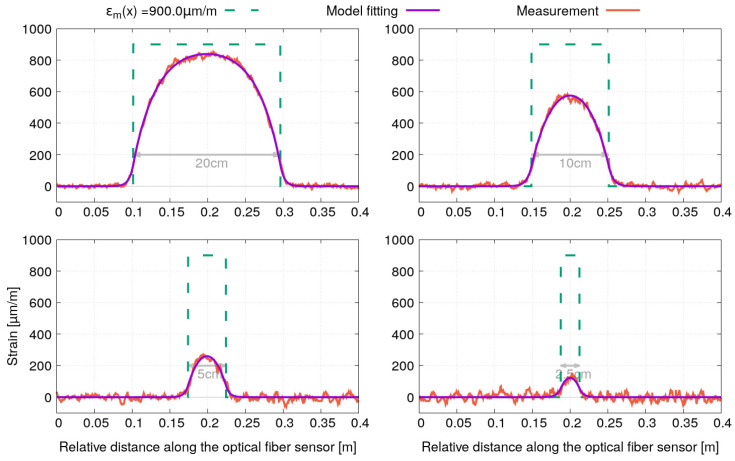
Fitting of the strain profiles obtained for the sample instrumented by optical fibers with acrylate coating under tensile test ($\sigma_{m}=180$ MPa).

Figure 9 and Figure 10 show the strain profiles and the model fitting for the optical fiber sensors with polyimide and acrylate coating, respectively. Note that for better readability, the strain profiles corresponding to each of the four bonding lengths have been shifted along the x-axis so that they are centered in a window of 40 cm width. A very good agreement can be observed between the fitted model and the strain profile measurements for both optical fiber sensors.

For model fitting, an implementation of the nonlinear least-squares Levenberg–Marquardt algorithm has been used to fit the parameters *a*, *b*, $\epsilon_{m}$, $\beta_{1}$ and $\beta_{2}$ of Equations (31a)–(31c). The values of these parameters obtained for the different bond lengths (L${}_{\mathrm{glued}}$) are presented in Table 2 and Table 3, respectively, for the optical fiber sensors with a polyimide and an acrylate coating. Before detailing the results of the fitted parameters, it is worth observing, particularly from Figure 10, that the strain profile measured by the optical fiber with an acrylate coating extends over a longer length (about 2 cm) than the bonding length, which confirms the new boundary conditions proposed in this paper.

Concerning the results of the model fitting, it can be observed from Table 2 and Table 3 that the bonding length has been very well estimated from the fit parameters *a* and *b* ($L_{\mathrm{glued}}=b-a$) for the two optical fiber sensors. The fit parameter $\epsilon_{m}$ has also been correctly estimated despite the increased error due to the measurement noise along the optical fiber. Note that for the acrylate coated fiber, the parameter $\epsilon_{m}$ had been fixed to 900μm/m for the strain profiles corresponding to 10 cm and 5 cm bond lengths in order for the fitting algorithm to converge. For the strain profiles corresponding to a bond length of 2.5 cm (the part closed to the end of the optical where the noise is the highest), three parameters *a*, *b* and $\epsilon_{m}$ have to be fixed as indicated in Table 3. From the model fitting, the most interesting fitting parameters are the strain lag parameters $\beta_{1}$ and $\beta_{2}$ since they are the key parameters for describing the strain transfer. As expected, the $\beta_{1}$ and $\beta_{2}$ values obtained for the optical fiber with a polyimide and acrylate coating are very different. They are higher for the optical fiber with a polyimide coating than the one with an acrylate coating.

**Figure 11:**
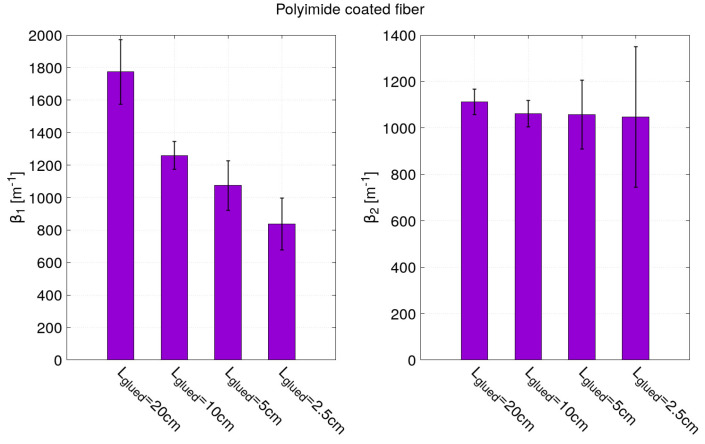
Fitting parameters $\beta_{1}$ and $\beta_{2}$ of the strain profiles obtained by optical fibers with polyimide coating.

**Figure 12:**
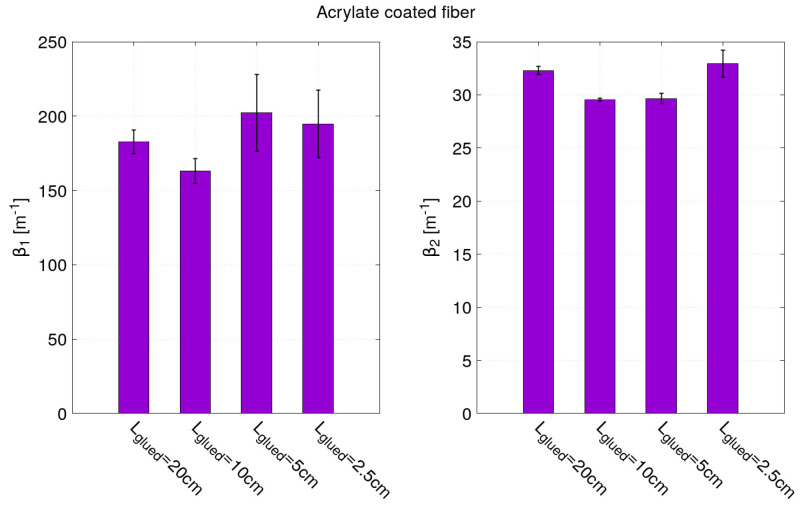
Fitting parameters $\beta_{1}$ and $\beta_{2}$ of the strain profiles obtained by optical fibers with acrylate coating.

As shown in Figure 11, the values of $\beta_{2}$ obtained for the four bond lengths of the optical fiber with a polyimide coating are close to 1050 m${}^{-1}$. The errors for fitting parameter $\beta_{2}$ can seem to increase as the glued length decreases. However, this error is more related to the level of noise (due in large part to vibrations of the hydraulic testing system). Indeed, the noise increases along the optical fiber and the smallest bonding lengths are localized near the end of the optical fiber. This phenomenon is more visible for the polyimide-coated fiber than for acrylate-coated fiber as it is characterized by a shorter strain transfer. The strain transfer of polyimide-coated fiber operates steeply over few millimeters contrary to a few centimeters for the acrylate-coated fiber. Given the spatial sampling of the measurements (0.65 mm), only five to six points are “useful” for the model fitting of the polyimide-coated fiber’s strain profile. This explains the difficulty of estimating $\beta_{1}$ and $\beta_{2}$ and the large dispersion of values obtained in Figure 11 for the polyimide-coated fiber. For the optical fiber with an acrylate coating, in Figure 12, values around 180 m${}^{-1}$ and 32 m${}^{-1}$ have been found for $\beta_{1}$ and $\beta_{2}$, respectively. These values of strain lag parameters are very much smaller than the ones obtained for the optical fiber with a polyimide coating. Thus, these results confirm that the higher the strain lag parameter is, the shorter the strain transfer distance is and, therefore, the more accurate the strain gradient measurements are. To further highlight the importance of strain transfer, in particular, in the presence of localized strain gradients, a non-uniform strain field case study is presented in the following part of this paper.

### 4.2. Non-Uniform Strain Field

#### 4.2.1. Description of the Experiment

**Figure 13:**
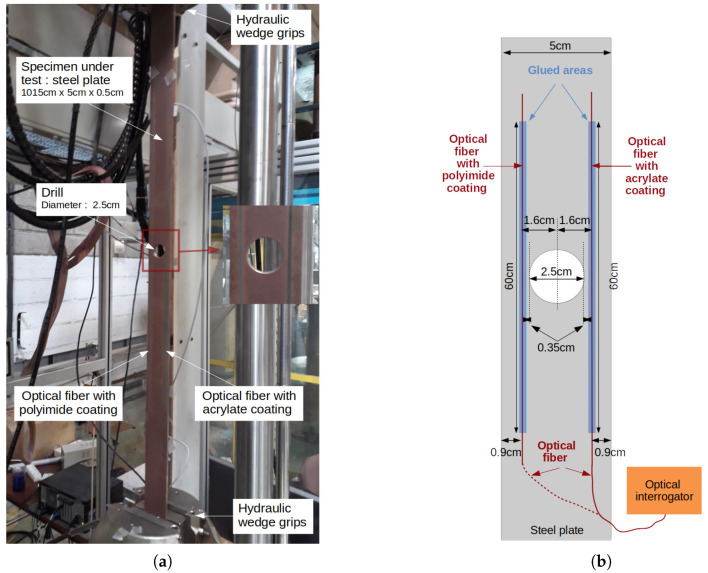
Experiment description of non-uniform strain field measurements. (**a**) Photography of the instrumented specimen under tensile test. (**b**) Schematic presentation of the specimen instrumentation.

In order to obtain a non-uniform strain field, a steel specimen (dimensions of the plate: 1015 cm × 5 cm × 5 mm; Young’s modulus: 200 GPa) with a hole (diameter: 2.5 cm) in the middle has been tested in tensile. Figure 13a shows the specimen in the wedge grips of servohydraulic testing system. A constant force of 8 kN (value low enough to avoid plasticity in the material) has been applied on the steel plate instrumented by two optical fibers. Similarly to the previous experiment, the same optical interrogator is used for distributed strain profiles measurements. In this case, both types of optical fibers were bonded over 60 cm length at 9 mm from the specimen edges as shown in Figure 13b. The tensile test has also been repeated twice and each optical fiber was interrogated separately in order to reduce the sensing length and to avoid having measurements that are too noisy due to the testing system vibrations.

#### 4.2.2. Results and Discussions

**Figure 14:**
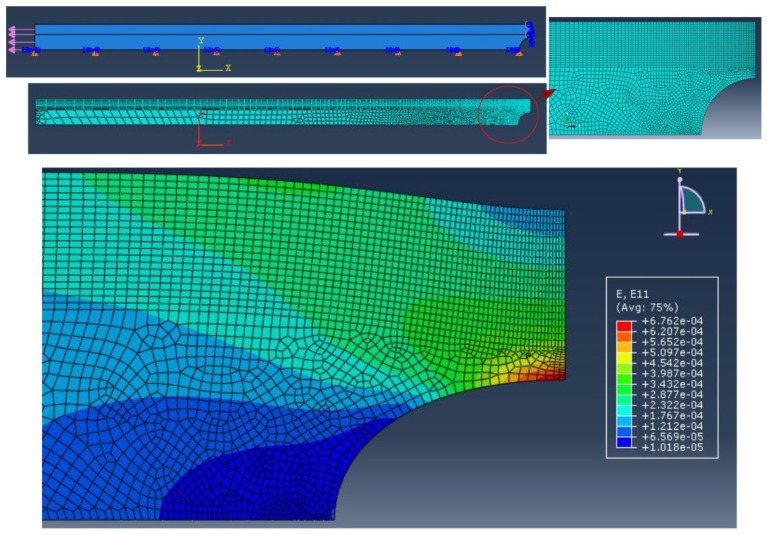
Finite element analysis of the sample (from ABAQUS by Dassault Systems${}^{®}$).

**Figure 15:**
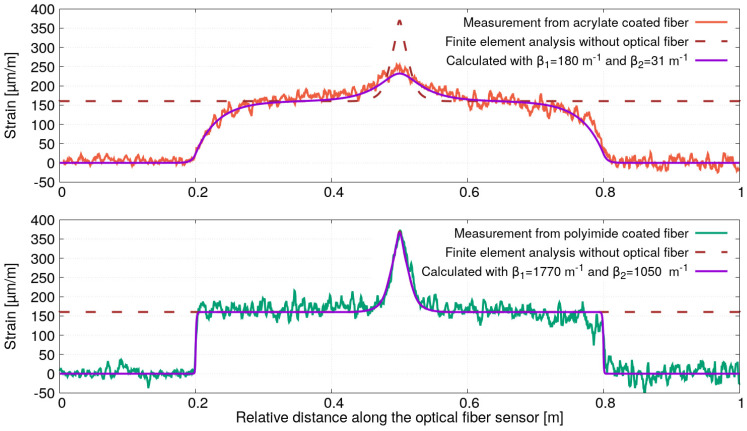
Comparisons of strain profile measured and calculated for the optical fibers with polyimide and acrylate coating.

In order to determine the strain profile $\epsilon_{m}\left(x\right)$ at the location of the optical fibers (at 9 mm from the edge of the steel plate), a finite element analysis has been performed. As shown in Figure 14, a symmetric quarter model was considered by using a plane strain computation in the elastic domain. Note that the optical fiber sensors have not been included in the simulation. Concerning the meshing, quad elements were used in the upper part of the specimen to extract the longitudinal strain profile at the location of the optical fiber on the entire length of the steel plate. The result of the finite element analysis is shown in dark-red dashed curves in both graphs of Figure 15. In the same figure, the strain profiles measured for the optical fibers (bonded on 60 cm length) having an acrylate (red curve in the upper graph) and a polyimide (green curve in the lower graph) coating are also plotted. As expected, the presence of a hole in the steel plate induces a peak of strain covering a distance of around 10 cm. However, it can be observed that the highest peak is obtained for the optical fiber with a polyimide coating. On both sides of this peak, the deformation along the specimen is constant, whereas the strain measurement decreases slowly for the optical fiber with an acrylate coating. The comparison of strain profile calculated from finite element analysis with the measurements shows once again the importance of strain transfer in distributed strain measurements, particularly in the presence of strain gradients. Ignoring this effect and using an optical fiber with a low value of the strain lag parameter can result in misinterpretation and, even worse, the underestimation of strain.

The differences in calculated and measured strain profiles can be more clearly understood by using the proposed strain transfer. For this reason, the strain profile in the core of both optical fibers has been calculated by using the proposed general solution (Equations (28a)–(28c)) with the strain lag parameters estimated in the experiment of uniform strain field. The strain profile obtained by finite element analysis has then been used to calculate the strain profile in the core of each of the two optical fibers with the following strain lag parameters: $\beta_{1}=180$ m${}^{-1}$ and $\beta_{2}=31$ m${}^{-1}$ for the optical fiber with acrylate coating and $\beta_{1}=1770$ m${}^{-1}$ and $\beta_{2}=1050$ m${}^{-1}$ for the optical fiber with polyimide coating. Note that the calculation of Equations (28a)–(28c) has been performed by using a numerical integration based on Simpson’s rule. A linear interpolation has also been used to obtained the strain profile $\epsilon_{m}\left(x\right)$ (calculated from the finite element analysis) at fixed spatial sampling accounting for the variable mesh sizes (small meshes in the area near the hole and large meshes in the area where the strain is almost constant). For comparison with the measured strain profiles, the results of the strain profile’s calculations are also plotted in Figure 15 (violet curves in the upper and lower graphs). A very good agreement between the measured and calculated strain profiles can be noticed. This result shows that the general solution proposed in this paper can be applied for the non-uniform strain profile $\epsilon_{m}\left(x\right)$.

It should be emphasized that the general solution is an efficient tool for determining the strain profiles in the core of an optical fiber provided that the correct strain lag parameters values are used. As a reminder, they depend both on the type of the optical fiber (or cable) used as a sensor and on its attachment to the surface or inside the material. For instance, the type of adhesive for surface-mounting or the procedure of surface preparation can result in different strain lag parameter values for the same optical fiber, and they are difficult to predict. Indeed, many parameters used in the Equation (11) of the strain lag parameter, for instance the interface stiffness parameters, are not well known and are difficult to measure. It is then recommended to perform tests on specimens as those performed in this paper to estimate the strain lag parameter for a given system: optical fiber/adhesive/surface or optical fiber/host material. It is worth also noting that finite element analysis that would include the optical fiber sensor requires the same parameters as those included in the expression of the strain lag parameter. However, due to the small dimensions of the optical fiber, a very fine meshing is generally required that may result in a high number of nodes and a long computation time. It could be also interesting to perform a finite element analysis without the optical fiber in order to estimate a non-uniform strain profile at the location of the optical fiber and then to calculate the strain profile in the core of the optical fiber. This should allow the estimation of the strain transfer effect on the strain profile measurements and, thus, to choose the best system optical fiber/adhesive/surface or optical fiber/host material. It is also important to highlight the fact that the measurements shown in this paper have been obtained with a high spatial resolution optical interrogator (0.65 mm gage pitch and 1.30 mm gage length according to manufacturer’s datasheet of ODISI-B). Measurements using lower spatial resolution instruments (for instance few centimeters or more) could result in additional measurement error due to an averaging effect. This effect is not taken into account in this paper since a high spatial resolution optical interrogator has been used.

## 5. Conclusions

In this paper, a general solution (Equations (28a)–(28c)) for determining the strain profile in the core of a distributed optical fiber sensor under any arbitrary strain fields is presented for the first time. This general solution permits the description of the strain transfer in distributed optical fiber sensors. It is based on a governing equation (Equation (12)) that is valid for embedded or surface attached optical fiber sensors, as discussed in Section 2.2, and includes a new set of boundary conditions (Equations (16a)–(18b)). This general solution has been validated experimentally by two experiments where the strain profiles measured with an optical interrogator possessing high spatial resolution (few millimeters) have been found in very good agreement with those obtained from the general solution.

It is worth remembering that the strain transfer phenomenon depends both on the type of optical fibers (or cables) used as a sensor and on its attachment to the surface or embedding in the material. The strain lag parameters defined in the general solution permits the completely characterization of this phenomenon. However, the estimations of these parameters from the geometrical and mechanical properties of the optical fiber sensors and the attachment conditions may often be inaccurate due to different parameters, such as interface stiffness parameters which are hardly known and difficult to measure. It is then recommended to perform more experimental tests to measure the strain lag parameters similar to those presented in Section 4.1.

Although this paper is focused on the presentation of the theoretical background and the validation of a general solution to determine the strain profile in the core of a distributed optical fiber sensor under any arbitrary strain fields with two types of optical fiber, further studies can be performed to investigate the strain transfer for a larger variety of optical cables used in distributed sensing and to test different methods to measure the strain lag parameters. It should be also interesting to study the effect of temperature, aging and fatigue on the strain lag parameter. These different studies could be the basis for a standardized characterization of the optical fiber/optical cables used in distributed sensing.

## Figures and Tables

**Table 1:** Mechanical properties and dimensions of the optical fiber components [13,22].

	Optical Fibers Components			
	Fiber’s Core	Optical Cladding	Coating	
Material	Silica	Silica	Acrylate	Polyimide
Diameter [μm]	9	125	250	160
Young’s modulus [GPa]	72	72	2.10${}^{-3}$	3
Shear modulus [GPa]	30	30	8.10${}^{-4}$	1.43

**Table 2:** Fitting parameters obtained for the sample instrumented by polyimide coated fibers.

OF with Polyimide Coating	*a* (m)	*b* (m)	$\epsilon_{m}$ (μm/m)	$\beta_{1}$ (m${}^{-1}$)	$\beta_{2}$ (m${}^{-1}$)
$L_{\mathrm{glued}}$ = 20 cm	0.0885 ± $1.10^{-4}$	0.2884 ± $1.10^{-4}$	908 ± 2	1774 ± 200	1112 ± 54
$L_{\mathrm{glued}}$ = 10 cm	0.1410 ± $1.10^{-4}$	0.2405 ± $1.10^{-4}$	911 ± 2	1259 ± 84	1061 ± 57
$L_{\mathrm{glued}}$ = 5 cm	0.1653 ± $2.10^{-4}$	0.2127 ± $2.10^{-4}$	913±6	1074 ± 152	1058 ± 148
$L_{\mathrm{glued}}$ = 2.5 cm	0.1587 ± $4.10^{-4}$	0.1844 ± $4.10^{-4}$	935 ± 15	837 ± 160	1047 ± 303

**Table 3:** Fitting parameters obtained for the sample instrumented by acrylate coated fibers.

OF with Acrylate Coating	*a* (m)	*b* (m)	$\epsilon_{m}$ (μm/m)	$\beta_{1}$ (m${}^{-1}$)	$\beta_{2}$ (m${}^{-1}$)
$L_{\mathrm{glued}}$ = 20 cm	0.1012 ± $3.10^{-4}$	0.2962 ± $3.10^{-4}$	905 ± 4	183 ± 8	32 ± 1
$L_{\mathrm{glued}}$ = 10 cm	0.1488 ± $4.10^{-4}$	0.2512 ± $4.10^{-4}$	fixed to 900	163 ± 9	30 ± 1
$L_{\mathrm{glued}}$ = 5 cm	0.1739 ± $8.10^{-4}$	0.2241 ± $8.10^{-4}$	fixed to 900	206 ± 27	30 ± 1
$L_{\mathrm{glued}}$ = 2.5 cm	fixed to 0.1875	fixed to 0.2125	fixed to 900	194 ± 22	33 ± 2

## Appendix A. Strain-Lag Parameter for a Three-Layers and Four-Layers System

In three-layers system, the strain-lag parameter β can be obtained by following the same method presented in the Section 2.1 and by only considering coating #1 in Figure 2. For more readability, the result of the calculation is expressed in the following form:

(A1) $$\beta^{2}=\frac{1}{\lambda }$$

where the following is the case.

(A2) $$\begin{array}{cc} \lambda = & \frac{E_{f}}{k_{p\;1/m}}\left(\frac{r_{p\;1}^{2}-r_{c}^{2}}{2\;r_{p\;1}}\;\frac{E_{p1}}{E_{f}}+\frac{r_{c}^{2}-r_{f}^{2}}{2\;r_{p\;1}}\;\frac{E_{c}}{E_{f}}+\frac{r_{f}^{2}}{2\;r_{p\;1}}\right)\\ \; & +\frac{E_{f}}{G_{p\;1}}\;\left[\left(\frac{r_{p\;1}^{2}-r_{c}^{2}}{4}-\frac{r_{c}^{2}}{2}\;\ln\left(\frac{r_{p\;1}}{r_{c}}\right)\right)\frac{E_{p\;1}}{E_{f}}+\left(\frac{r_{c}^{2}-r_{f}^{2}}{2}\;\frac{E_{c}}{E_{f}}+\frac{r_{f}^{2}}{2}\right)\;\ln\left(\frac{r_{p\;1}}{r_{c}}\right)\right]\\ \; & +\frac{E_{f}}{k_{c}}\left(\frac{r_{c}^{2}-r_{f}^{2}}{2\;r_{c}}\;\frac{E_{c}}{E_{f}}+\frac{r_{f}^{2}}{2\;r_{c}}\right)\\ \; & +\frac{E_{f}}{G_{c}}\;\left[\left(\frac{r_{c}^{2}-r_{f}^{2}}{4}-\frac{r_{f}^{2}}{2}\;\ln\left(\frac{r_{c}}{r_{f}}\right)\right)\frac{E_{c}}{E_{f}}+\frac{r_{f}^{2}}{2}\;\ln\left(\frac{r_{c}}{r_{f}}\right)\right] \end{array}$$

In the same way, the strain-lag parameter β for four-layers system can determined by considering only the coatings #1 and #2 in Figure 2. The calculation gives us the following.

(A3) $$\begin{array}{cc} \lambda = & \frac{E_{f}}{k_{p\;2/m}}\left(\frac{r_{p2}^{2}-r_{p1}^{2}}{2\;r_{p2}}\;\frac{E_{p2}}{E_{f}}+\frac{r_{p1}^{2}-r_{c}^{2}}{2\;r_{p2}}\;\frac{E_{p1}}{E_{f}}+\frac{r_{c}^{2}-r_{f}^{2}}{2\;r_{p2}}\;\frac{E_{c}}{E_{f}}+\frac{r_{f}^{2}}{2\;r_{p2}}\right)\\ \; & +\frac{E_{f}}{G_{p\;2}}\;\left[\left(\frac{r_{p\;2}^{2}-r_{p\;1}^{2}}{4}-\frac{r_{p\;1}^{2}}{2}\;\ln\left(\frac{r_{p\;2}}{r_{p\;1}}\right)\right)\frac{E_{p\;2}}{E_{f}}\right.+\\ \; & \left.\left(\frac{r_{p\;1}^{2}-r_{c}^{2}}{2}\;\frac{E_{p\;1}}{E_{f}}+\frac{r_{c}^{2}-r_{f}^{2}}{2}\;\frac{E_{c}}{E_{f}}+\frac{r_{f}^{2}}{2}\right)\;\ln\left(\frac{r_{p\;2}}{r_{p\;1}}\right)\right]\\ \; & +\frac{E_{f}}{k_{p\;1/2}}\left(\frac{r_{p\;1}^{2}-r_{c}^{2}}{2\;r_{p\;1}}\;\frac{E_{p1}}{E_{f}}+\frac{r_{c}^{2}-r_{f}^{2}}{2\;r_{p\;1}}\;\frac{E_{c}}{E_{f}}+\frac{r_{f}^{2}}{2\;r_{p\;1}}\right)\\ \; & +\frac{E_{f}}{G_{p\;1}}\;\left[\left(\frac{r_{p\;1}^{2}-r_{c}^{2}}{4}-\frac{r_{c}^{2}}{2}\;\ln\left(\frac{r_{p\;1}}{r_{c}}\right)\right)\frac{E_{p\;1}}{E_{f}}+\left(\frac{r_{c}^{2}-r_{f}^{2}}{2}\;\frac{E_{c}}{E_{f}}+\frac{r_{f}^{2}}{2}\right)\;\ln\left(\frac{r_{p\;1}}{r_{c}}\right)\right]\\ \; & +\frac{E_{f}}{k_{c}}\left(\frac{r_{c}^{2}-r_{f}^{2}}{2\;r_{c}}\;\frac{E_{c}}{E_{f}}+\frac{r_{f}^{2}}{2\;r_{c}}\right)\\ \; & +\frac{E_{f}}{G_{c}}\;\left[\left(\frac{r_{c}^{2}-r_{f}^{2}}{4}-\frac{r_{f}^{2}}{2}\;\ln\left(\frac{r_{c}}{r_{f}}\right)\right)\frac{E_{c}}{E_{f}}+\frac{r_{f}^{2}}{2}\;\ln\left(\frac{r_{c}}{r_{f}}\right)\right] \end{array}$$

## Appendix B. Particular Solution of Equation (12)

In order to find a particular solution of Equation (12), it is assumed that the solution can be written as follows:

(A4) $$\epsilon_{f}^{p}\left(x\right)=A\left(x\right)\;\exp\left(\beta \;x\right)+B\left(x\right)\;\exp\left(-\beta \;x\right)$$

where A(x) and B(x) are unknown functions that we are looking for. To perform this, the derivative of (A4) is first calculated.

(A5) $$\epsilon_{f}^{p\;\prime }\left(x\right)=A^{\prime }\left(x\right)\;\exp\left(\beta \;x\right)+\beta \;A\left(x\right)\;\exp\left(\beta \;x\right)+B^{\prime }\left(x\right)\;\exp\left(-\beta \;x\right)-\beta \;B\left(x\right)\;\exp\left(-\beta \;x\right)$$

The following condition is imposed.

(A6) $$A^{\prime }\left(x\right)\;\exp\left(\beta \;x\right)+B^{\prime }\left(x\right)\;\exp\left(-\beta \;x\right)=0$$

Consequently, Equation (A5) is reduced to the following.

(A7) $$\epsilon_{f}^{p\;\prime }\left(x\right)=\beta \;A\left(x\right)\;\exp\left(\beta \;x\right)-\beta \;B\left(x\right)\;\exp\left(-\beta \;x\right)$$

The derivative of Equation (A7) provides the following.

(A8) $$\epsilon_{f}^{p\;\prime \prime }\left(x\right)=A^{\prime }\left(x\right)\;\beta \;\exp\left(\beta \;x\right)+\beta^{2}\;A\left(x\right)\;\exp\left(\beta \;x\right)-B^{\prime }\left(x\right)\;\beta \;\exp\left(-\beta \;x\right)+\beta^{2}\;B\left(x\right)\;\exp\left(-\beta \;x\right)$$

By introducing (A7) and (A8) in (12), the following equation can be obtained.

(A9) $$A^{\prime }\left(x\right)\;\beta \;\exp\left(\beta \;x\right)-B^{\prime }\left(x\right)\;\beta \;\exp\left(-\beta \;x\right)=-\beta^{2}\;\epsilon_{m}\left(x\right)$$

By using the condition (A6), the following equation on A(x) can be deduced.

(A10) $$A^{\prime }\left(x\right)=-\frac{\beta }{2}\;\epsilon_{m}\left(x\right)\;\exp\left(-\beta \;x\right)$$

In the same manner, an equation on B(x) can also be obtained.

(A11) $$B^{\prime }\left(x\right)=\frac{\beta }{2}\;\epsilon_{m}\left(x\right)\;\exp\left(\beta \;x\right)$$

At this point, we must note the following:

(A12) $$\int_{0}^{x}A^{\prime }\left(x\prime \right)\;dx\prime =A\left(x\right)-A\left(0\right)$$

and

(A13) $$\int_{0}^{x}B^{\prime }\left(x\prime \right)\;dx\prime =B\left(x\right)-B\left(0\right)$$

Moreover, the following conditions are supposed to be verified:

(A14a) $$\begin{array}{cc} A(0)= & 0 \end{array}$$

(A14b) $$\begin{array}{cc} B(0)= & 0 \end{array}$$

such that the following can be obtained.

(A15a) $$\begin{array}{cc} A(x)= & \int_{0}^{x}A^{\prime }\left(x\prime \right)\;dx\prime \end{array}$$

(A15b) $$\begin{array}{cc} B(x)= & \int_{0}^{x}B^{\prime }\left(x\prime \right)\;dx\prime \end{array}$$

By incorporating (A15a) and (A15b) in Equation (A4), $\epsilon_{f}\left(x\right)$can be expressed as follows.

(A16) $$\epsilon_{f}^{p}\left(x\right)=\int_{0}^{x}A^{\prime }\left(x\prime \right)\;dx\prime \;\exp\left(\beta \;x\right)+\int_{0}^{x}B^{\prime }\left(x\prime \right)\;dx\prime \;\exp\left(-\beta \;x\right)$$

In this last expression, $A^{\prime }\left(x\prime \right)$ and $A^{\prime }\left(x\prime \right)$ can be replaced by the relations (A10) and (A11).

(A17) $$\epsilon_{f}^{p}\left(x\right)=-\frac{\beta }{2}\int_{0}^{x}\epsilon_{m}\left(x\prime \right)\;\exp\left(-\beta \;x\prime \right)\;dx\prime \;\exp\left(\beta \;x\right)+\frac{\beta }{2}\int_{0}^{x}\epsilon_{m}\left(x\prime \right)\;\exp\left(\beta \;x\prime \right)\;dx\prime \;\exp\left(-\beta \;x\right)$$

By rearranging Equation (A17), the expression of a particular solution of Equation (12) can be written as follows.

(A18) $$\epsilon_{f}^{p}\left(x\right)=-\beta \int_{0}^{x}\epsilon_{m}\left(x\prime \right)\;\sinh\left(\beta \left(x-x\prime \right)\right)\;dx\prime$$

## Appendix C. Derivation of the Parameters *A*_1_, *B*_1_, *A*_2_, *B*_2_, *A*_3_ and ^B^_3_

✧ Case: 0≤x≤a

Using the boundary condition (16a) ($\epsilon_{f\;1}\left(0\right)=0$), it can be deduced immediately from Equation (20) that $A_{1}$ and $B_{1}$ must verify the following equation.

(A19) $$A_{1}=-B_{1}$$

Consequently, Equation (20) can be rewritten as follows.

(A20) $$\epsilon_{f\;1}\left(x\right)=2\;A_{1}\;\sinh\left(\beta_{1}\;x\right)$$

As $\epsilon_{f\;1}\left(a\right)=\epsilon_{a}$, $A_{1}$ is given by the following.

(A21) $$A_{1}=\frac{\epsilon_{a}}{2\;\sinh(\beta_{1}\;a)}$$

✧ Case: a≤x≤b

As $\epsilon_{f\;2}\left(a\right)=\epsilon_{a}$ and $\epsilon_{f\;2}\left(b\right)=\epsilon_{b}$ and by noticing that $\epsilon_{f\;2}^{p}\left(a\right)=0$, the contants $A_{2}$ and $B_{2}$ must verify the following new set of equations.

(A22a) $$\begin{array}{cc} A_{2}\;\exp\left(\beta_{2}\;a\right)+B_{2}\;\exp\left(-\beta_{2}\;a\right) & =\epsilon_{a} \end{array}$$

(A22b) $$\begin{array}{cc} A_{2}\;\exp\left(\beta_{2}\;b\right)+B_{2}\;\exp\left(-\beta_{2}\;b\right)+\epsilon_{f\;2}^{p}\left(b\right) & =\epsilon_{b} \end{array}$$

✧ Case: b≤x≤L

As $\epsilon_{f\;3}\left(b\right)=\epsilon_{b}$ and with the boundary condition (16a) ($\epsilon_{f\;3}\left(L\right)=0$), $A_{3}$ and $B_{3}$ must verify the following new set of equations.

(A23a) $$\begin{array}{cc} A_{3}\;\exp\left(\beta_{1}\;b\right)+B_{3}\;\exp\left(-\beta_{1}\;b\right)+\epsilon_{f\;3}^{p}\left(b\right) & =\epsilon_{b} \end{array}$$

(A23b) $$\begin{array}{cc} A_{3}\;\exp\left(\beta_{1}\;L\right)+B_{3}\;\exp\left(-\beta_{1}\;L\right)+\epsilon_{f\;3}^{p}\left(L\right) & =0 \end{array}$$

From (A23b), it becomes immediately clear that $B_{3}$ is given by the following.

(A24) $$B_{3}=-\left(A_{3}\;\exp\left(2\;\beta_{1}\;L\right)+\epsilon_{f\;3}^{p}\left(L\right)\;\exp\left(\beta_{1}\;L\right)\right)$$

Consequently, Equation (24) can be rewritten as follows:

(A25) $$\epsilon_{f\;3}\left(x\right)=A_{3}\;\left(\exp\left(\beta_{1}\;x\right)-\exp(\beta_{1}\left(2\;L-x\right)\right)-\epsilon_{f\;3}^{p}\left(L\right)\;\exp(\beta_{1}\left(L-x\right)+\epsilon_{f\;3}^{p}\left(x\right)$$

and the constant $A_{3}$ can be expressed for x=b as the following.

(A26) $$A_{3}=\frac{\epsilon_{f\;3}^{p}\left(L\right)\;\exp\left(\beta_{1}\left(L-b\right)\right)-\epsilon_{f\;3}^{p}\left(b\right)+\epsilon_{b}}{\exp\left(\beta_{1}\;x\right)-\exp\left(\beta_{1}\;\left(2\;L-b\right)\right)}$$

From Equations (A21) and (A22a), the quantity $\epsilon_{a}$ can be removed which gives us the following.

(A27) $$2\;A_{1}\;\sinh\left(\beta_{1}\;a\right)-A_{2}\;\exp\left(\beta_{2}\;a\right)-B_{2}\;\exp\left(-\beta_{2}\;a\right)=0$$

In the same way, from Equations (A22a), (A23a) and (A24), the quantity $\epsilon_{b}$ can be removed, which results in the following relation.

(A28) $$\begin{array}{cc} \; & A_{2}\;\exp\left(\beta_{2}\;b\right)+B_{2}\;\exp\left(-\beta_{2}\;b\right)+\epsilon_{f\;2}^{p}\left(b\right)-A_{3}\;\left(\exp\left(\beta_{1}\;b\right)-\exp\left(2\;\beta_{1}\;L\right)\right)+\\ \; & \epsilon_{f\;3}^{p}\left(L\right)\;\exp\left(\beta_{1}\left(L-b\right)\right)-\epsilon_{f\;3}^{p}\left(b\right)=0 \end{array}$$

By calculating the derivatives of $\epsilon_{f\;1}\left(x\right)$, $\epsilon_{f\;2}\left(x\right)$ and $\epsilon_{f\;3}\left(x\right)$ from Equations (A20), (22) and (A25) (and noting that ${\left.\frac{d\epsilon_{f\;2}^{p}\left(x\right)}{dx}\right|}_{x=a}=0$) and by using the conditions (17a) and (17b), the two following equations can be obtained:

(A29) $$2\;\beta_{1}\;A_{1}\;\cosh\left(\beta_{1}\;a\right)-\beta_{2}\;A_{2}\;\exp\left(\beta_{2}\;a\right)+\beta_{2}\;B2\exp\left(-\beta_{2}\;a\right)=0$$

and

(A30) $$\begin{array}{cc} \; & \beta_{2}\;A_{2}\;\exp\left(\beta \;b\right)-\beta_{2}\;B_{2}\exp\left(-\beta \;b\right)+{\left.\frac{d\epsilon_{f\;2}^{p}\left(x\right)}{dx}\right|}_{x=b}-\beta_{1}\;A_{3}\;\left(\exp\left(\beta_{1}\;b\right)+\exp\left(\beta_{1}\;\left(2\;L-b\right)\right)\right)-\\ \; & \beta_{1}\;\epsilon_{f\;3}^{p}\left(L\right)\;\exp\left(\beta_{1}\;\left(L-b\right)\right)-{\left.\frac{d\epsilon_{f\;3}^{p}\left(x\right)}{dx}\right|}_{x=b}=0. \end{array}$$

In Equations (A27)–(A30), $A_{1}$, $A_{2}$, $B_{2}$ and $A_{3}$ are unknown constants that can be determined by rewriting the latter equations in the matrix form given by Equation (25). By applying Cramer’s rule, the parameters $A_{1}$, $A_{2}$, $B_{2}$ and $A_{3}$ are given by the following:

(A31a) $$\begin{array}{cc} \;A_{1}= & \frac{\beta_{2}}{K\;\exp(\beta_{1}\;L)}\;[\left(R_{4}+\beta_{1}\;R_{3}\right)\;\exp\left(\beta_{1}\;\left(2\;L-b\right)\right)-\left(R_{4}-\beta_{1}\;R_{3}\right)\;\exp\left(\beta_{1}\;b\right)] \end{array}$$

(A31b) $$\begin{array}{cc} A_{2}= & \frac{1}{2\;K\;\exp(\beta_{1}\;L)}\;\{\left(\beta_{2}-\beta_{1}\right)\;\left[\left(R_{4}-\beta_{1}\;R_{3}\right)\;\exp\left(\beta_{1}\;\left(b-a\right)-\beta_{2}\;a\right)-\right.\\ \; & \;\left(R_{4}+\beta_{1}\;R_{3}\right)\;\exp\left(\beta_{1}\;\left(2\;L-b-a\right)-\beta_{2}\;a\right)]\\ \; & \;\left(\beta_{1}+\beta_{2}\right)\;\left[\left(R_{4}+\beta_{1}\;R_{3}\right)\;\exp\left(\beta_{1}\;\left(2\;L-b+a\right)-\beta_{2}\;a\right)-\right.\\ \; & \;\left.\left(R_{4}-\beta_{1}\;R_{3}\right)\;\exp\left(\beta_{1}\;\left(b+a\right)-\beta_{2}\;a\right)\right]\} \end{array}$$

(A31c) $$\begin{array}{cc} B_{2}= & \frac{1}{2\;K\;\exp(\beta_{1}\;L)}\;\left\{\left(\beta_{2}-\beta_{1}\right)\;\left[\left(\beta_{1}\;R_{3}-R_{4}\right)\;\exp\left(\beta_{1}\;\left(a+b\right)+\beta_{2}\;a\right)+\right.\right.\\ \; & \;\left.\left(R_{4}+\beta_{1}\;R_{3}\right)\;\exp\left(\beta_{1}\;\left(2\;L-b+a\right)+\beta_{2}\;a\right)\right]\\ \; & \;\left(\beta_{1}+\beta_{2}\right)\;\left[\left(R_{4}-\beta_{1}\;R_{3}\right)\;\exp\left(\beta_{1}\;\left(b-a\right)+\beta_{2}\;a\right)-\right.\\ \; & \;\left.\left.\left(R_{4}+\beta_{1}\;R_{3}\right)\;\exp\left(\beta_{1}\;\left(2\;L-b-a\right)+\beta_{2}\;a\right)\right]\right\} \end{array}$$

(A31d) $$\begin{array}{cc} A_{3}= & \frac{1}{2\;K\;\exp(\beta_{1}\;L)}\;\left\{\left(\beta_{2}-\beta_{1}\right)\;\left[\left(R_{4}-\beta_{2}\;R_{3}\right)\;\exp\left(\beta_{2}\;\left(b-a\right)-\beta_{1}\;a\right)-\right.\right.\\ \; & \;\left.\left(R_{4}+\beta_{2}\;R_{3}\right)\;\exp\left(\beta_{2}\;\left(a-b\right)+\beta_{1}\;a\right)\right]\\ \; & \;\left(\beta_{1}+\beta_{2}\right)\;\left[\left(R_{4}+\beta_{2}\;R_{3}\right)\;\exp\left(\beta_{2}\;\left(a-b\right)-\beta_{1}\;a\right)-\right.\\ \; & \;\left.\left.\left(R_{4}-\beta_{2}\;R_{3}\right)\;\exp\left(\beta_{2}\;\left(b-a\right)+\beta_{1}\;a\right)\right]\right\} \end{array}$$

where $R_{3}$ and $R_{4}$ are given by Equations (26c) and (26d) and the expression of coefficient *K* is as follows.

(A32) $$\begin{array}{cc} K= & {\left(\beta_{1}-\beta_{2}\right)}^{2}\;\sinh\left(\left(\beta_{1}+\beta_{2}\right)\;\left(b-a\right)-\beta_{1}\;L\right)\\ \; & -{\left(\beta_{1}+\beta_{2}\right)}^{2}\;\sinh\left(\left(\beta_{1}-\beta_{2}\right)\;\left(b-a\right)-\beta_{1}\;L\right)\\ \; & +2\;\left(\beta_{1}^{2}-\beta_{2}^{2}\right)\;\sinh\left(\beta_{2}\;\left(b-a\right)\right)\;\cosh\left(\beta_{1}\;\left(a+b-L\right)\right) \end{array}$$
